# Epidermal Growth Factor Receptor Inhibition Is Protective in Hyperoxia-Induced Lung Injury

**DOI:** 10.1155/2022/9518592

**Published:** 2022-09-20

**Authors:** Zachary M. Harris, Ying Sun, John Joerns, Brian Clark, Buqu Hu, Asawari Korde, Lokesh Sharma, Hyeon Jun Shin, Edward P. Manning, Lindsey Placek, Derya Unutmaz, Gail Stanley, Hyung Chun, Maor Sauler, Govindarajan Rajagopalan, Xuchen Zhang, Min-Jong Kang, Jonathan L. Koff

**Affiliations:** ^1^Section of Pulmonary, Critical Care, and Sleep Medicine; Department of Internal Medicine, Yale University School of Medicine, New Haven, Connecticut, USA 06510; ^2^Division of Pulmonary and Critical Care; Department of Internal Medicine, UT Southwestern Medical Center, Dallas, Texas, USA 75390; ^3^VA Connecticut Healthcare System, West Haven, CT, USA; ^4^The Jackson Laboratory for Genomic Medicine, Farmington, Connecticut 06032, USA; ^5^Section of Cardiovascular Medicine; Department of Internal Medicine, Yale University School of Medicine, New Haven, Connecticut, USA 06510; ^6^Department of Pathology, Yale University School of Medicine, New Haven, Connecticut, USA 06510

## Abstract

**Aims:**

Studies have linked severe hyperoxia, or prolonged exposure to very high oxygen levels, with worse clinical outcomes. This study investigated the role of epidermal growth factor receptor (EGFR) in hyperoxia-induced lung injury at very high oxygen levels (>95%).

**Results:**

Effects of severe hyperoxia (100% oxygen) were studied in mice with genetically inhibited EGFR and wild-type littermates. Despite the established role of EGFR in lung repair, EGFR inhibition led to improved survival and reduced acute lung injury, which prompted an investigation into this protective mechanism. Endothelial EGFR genetic knockout did not confer protection. EGFR inhibition led to decreased levels of cleaved caspase-3 and poly (ADP-ribosyl) polymerase (PARP) and decreased terminal dUTP nick end labeling- (TUNEL-) positive staining in alveolar epithelial cells and reduced ERK activation, which suggested reduced apoptosis *in vivo*. EGFR inhibition decreased hyperoxia (95%)-induced apoptosis and ERK in murine alveolar epithelial cells *in vitro*, and CRISPR-mediated EGFR deletion reduced hyperoxia-induced apoptosis and ERK in human alveolar epithelial cells *in vitro*. *Innovation*. This work defines a protective role of EGFR inhibition to decrease apoptosis in lung injury induced by 100% oxygen. This further characterizes the complex role of EGFR in acute lung injury and outlines a novel hyperoxia-induced cell death pathway that warrants further study.

**Conclusion:**

In conditions of severe hyperoxia (>95% for >24 h), EGFR inhibition led to improved survival, decreased lung injury, and reduced cell death. These findings further elucidate the complex role of EGFR in acute lung injury.

## 1. Introduction

Administration of supplemental oxygen is a lifesaving treatment and an irreplaceable component to supportive care for acute respiratory distress syndrome (ARDS) [1]. However, the deleterious effects of prolonged exposure to very high oxygen concentrations (>95%) in animals was identified over two hundred years ago [2]. Toxicity from protracted exposure to very high oxygen concentrations (severe hyperoxia) has been characterized in humans [3], and animal models of severe hyperoxia have helped to elucidate the pathogenesis and clinical consequences of this lung injury [4, 5]. Thus, hyperoxia-induced lung injury serves as an experimental model of sterile acute lung injury and lung repair following injury [6]. In addition to longstanding scientific knowledge of toxicity from severe hyperoxia, clinical evidence has confirmed the association of hyperoxia with worse clinical outcomes in critically ill patients [7, 8]. Severe hyperoxia is also associated with increased infection [9], exacerbates ventilator-induced lung injury (VILI) [10], and is associated with worse outcomes in cardiovascular and cerebrovascular disease [11]. Recognition of severe hyperoxia's clinical relevance has focused attention on the study of hyperoxia-induced lung injury. In addition, further elucidating the mechanisms of hyperoxia-induced lung injury may lead to the identification of important treatment strategies for lung injury and ARDS where high oxygen levels are consistently used as supportive therapy [1].

The pathogenesis of hyperoxia-induced lung injury involves generation of reactive oxygen species (ROS) that results in epithelial and endothelial cell injury [2, 12]. Severe hyperoxia activates mitogen-activated protein (MAP) kinases [12, 13], including extracellular signal-regulated kinase 1 and 2 (ERK1/2) [14–17], c-Jun N-terminal kinase 1 and 2 (JNK1/2) [18], and protein 38 (p38) [17]. Downstream from MAP kinase signaling pathways are diverse cellular effects that include both prosurvival and cell death pathways, such as apoptosis and necrosis [13]. Furthermore, the phosphatidylinositol 3-kinase (PI3K (PI3))/Ak strain transforming (AKT)) pathway, which is associated with cell survival [19], has been implicated in protective epithelial cell mechanisms in severe hyperoxia [15, 16].

Epidermal growth factor receptor (EGFR) is a transmembrane tyrosine kinase receptor with pleiotropic cellular effects [20], yet its role in hyperoxia-induced lung injury is not clearly defined. EGFR activation has been implicated in respiratory epithelial repair following naphthalene-induced lung injury in an animal model [21, 22]. Additionally, at the cellular level, EGFR is involved in alveolar epithelial cell proliferation [23, 24], wound repair in airway [25] and alveolar epithelial cells [26], alveolar epithelial cell migration [24, 26, 27], and restoration of epithelial cell barrier function after injury [28], all processes which provide a benefit in lung injury. However, EGFR activation has also been characterized as harmful in VILI [29] and lipopolysaccharide- (LPS-) induced lung injury [30]. EGFR also plays a deleterious role in other pulmonary conditions, such as asthma [31], viral infection [32–34], and pulmonary fibrosis [35, 36]. Consistent with the complex role of EGFR in lung injury, EGFR activation has been shown to have diverse effects on terminal cellular events. EGFR activation has been shown to be proapoptotic in epithelial breast adenocarcinoma cells [37] and in mammary tumor cells [38]. Conversely, EGFR activation has been shown to suppress epithelial cell apoptosis in an LPS model of acute lung injury [39], and EGFR inhibition with a tyrosine kinase inhibitor promoted airway epithelial cell apoptosis in a tumor necrosis factor- (TNF-) induced model of acute lung injury [40]. In addition, EGFR activation is known to trigger the PI3/AKT pathway, which has been shown to regulate antioxidant transcriptional responses in alveolar epithelial cells in response to severe hyperoxia [16].

Based on the known positive effects of EGFR signaling in lung injury, we initially hypothesized that EGFR inhibition would lead to increased susceptibility to severe hyperoxia. To test this hypothesis, genetically modified mice (EGFR^Wa5/+^ mice) with reduced EGFR activity and wild-type (WT) littermates were subjected to excessively high oxygen levels (100% for >24 h). We were surprised to find that decreased EGFR activity was protective, and this result prompted an investigation into the mechanism(s) whereby decreased EGFR activity is beneficial in hyperoxia-induced lung injury. Subsequent experiments found that EGFR^Wa5/+^ mice had decreased pulmonary cell death in severe hyperoxia (100% oxygen). *In vitro* experiments confirmed that EGFR inhibition reduces alveolar epithelial apoptosis in severe hyperoxia. These results are important because they further characterize the complex role of EGFR in acute lung injury and define a cell death pathway involved in excessive oxygen toxicity that may be amenable to therapeutic modulation.

## 2. Materials and Methods

### 2.1. Mice

EGFR-deficient mice containing the Wa5 Egfr allele (EGFR^Wa5/+^ mice) and mice carrying a conditional allele for EGFR (EGFR^fl/fl^) were a generous gift from Dr. David Threadgill at Texas A&M College of Medicine. The Egfr Wa5 allele contains a missense mutation within the EGFR coding region that results in decreased EGFR activity [41]. Thus, Egfr Wa5 functions as a hypomorphic Egfr allele. The genetic background of EGFR^Wa5/+^ mice is BALB/c, C3H, and C57Bl/6J [41]. EGFR^fl/fl^ mice were crossed with VE-Cadherin-Cre recombinase transgenic mice to produce mice with endothelial-specific EGFR knockout (EGFR^EndoKO^) [42]. The genetic background of EGFR^EndoKO^ transgenic mice is C57BL/6J, which have been backcrossed for six generations [42, 43], and their phenotypic characterization has been described elsewhere [42].

### 2.2. Severe Hyperoxia Exposures

Adult eight- to ten-week-old mice were exposed to 100% oxygen delivered continuously in a plexiglass hyperoxia chamber [44]. For survival experiments, mice (*n* = 6 per group) were closely monitored, and time of death was recorded. For timed exposures, mice (*n* = 6 per group) were exposed to 100% oxygen continuously for 24 h (mild injury), 48 h (moderate injury), and 72 h (severe injury). Lung specimens were taken for histology, RNA, protein, and immunohistochemistry analysis (described below). All protocols were reviewed and approved by Yale University IACUC.

For cell culture experiments, murine lung epithelial 12 (MLE12) cells (American Type Culture Collection (ATCC), Manassas, CA, USA; item # CRL-2100) and A-549 (ATCC; item # CCL-185) were used. MLE12 cells exhibit characteristics of type-II distal respiratory epithelium [45] and were maintained in Dulbecco's modified Eagle's medium (DMEM)/F-12 (50/50 mix) (Thermo Fisher Scientific, Waltham, MA, USA; catalog # 11330057) supplemented with 0.1% fetal bovine serum (FBS) (Sigma, St. Louis, MO, USA; catalog # F2442), penicillin-streptomycin (Thermo Fisher; catalog #15140122), and L-glutamine (Thermo Fisher; catalog #25030149). A-549 cells are an established cell line that exhibit characteristics of type-II alveolar epithelial cells [46]. CRISPR modification of A-549 cells was accomplished via the following. The sequence of gRNA used for EGFR knockout, GAATTCGCTCCACTGTGTTG, was designed and cloned into the LentiCRISPR v2 vector as previously described [47]. Lentivirus pseudotyped with the vesicular stomatitis virus G protein envelope was generated using HEK293T cells [47]. A549-Dual™ cells (Invivogen, Location) were transduced with the EGFR-targeting CRISPR lentivirus and selected with 1 *μ*g/mL puromycin (Thermo Fisher; catalog #1113803) for 4 days, or until all noninfected cells were dead. Cells were stained for EGFR expression using an adenomatous polyposis coli (APC) anti-human EGFR Ab and analyzed via flow cytometry. EGFR CRISPR (A-549^EGFRko^) cells showed 99% EGFR knockdown compared to control cells transduced with an empty vector (A-549^CRISPR^) (data not shown). A-549 cells (A-549 control, A-549^CRISPR^, and A-549^EGFRko^) were maintained in DMEM (Thermo Fisher; catalog #11966025) supplemented with 0.1% FBS (Sigma), penicillin-streptomycin (Thermo Fisher), and L-glutamine (Thermo Fisher). In addition, puromycin was added to culture medium (maintenance culture medium) of A-549^EGFRko^ and A-549^CRISPR^ to select for cells containing CRISPR modification. Cells were cultured at 37°C containing 5% carbon dioxide.

For *in vitro* hyperoxia exposure, all experiments were conducted using a monolayer of confluent cells to avoid cell density variability between control cells and those exposed to hyperoxia [48]. Cells were cultured 24 hours prior to hyperoxia exposure [48]. For MLE12 cells, experiments were performed in DMEM/F-12 medium not containing FBS (i.e., serum-free medium). For A-549 cells, including cells modified via CRISPR, DMEM not containing FBS was used. Cell cultures were exposed to hyperoxia (95% oxygen/5% carbon dioxide) in a tightly sealed modular exposure chamber (Billup-Rothberg, Del Mar, CA, USA; item #MIC-101) for predetermined time points. For EGFR inhibition, selective tyrosine kinase EGFR inhibitors (1) gefitinib (Selleck, Houston, TX, USA; catalog #S1025), and (2) Ag-1478 (EMD Millipore, Billerica, MA, USA; item #658552) were used. Ag-9 (Millipore; item #658390), a tyrosine kinase analog, was used for a negative control. Dimethyl sulfoxide (DMSO) (Sigma-Aldrich, Saint Louis, MO, USA; catalog #D8418), the vehicle for gefitinib, was also used as a negative control. The apoptosis inducer ABT-263 (Selleck; catalog #S1001) was used as a positive control for apoptosis. Cell supernatants were collected for lactate dehydrogenase (LDH) analysis. Cell lysates were collected for protein analysis.

### 2.3. Measurements of Lung Injury

BAL fluid was assessed 24, 48, and 72 h after severe hyperoxia exposure [49]. The trachea was cannulated and perfused with two aliquots of 0.9% cold saline. BAL was separated via centrifugation. BAL fluid total cell count was determined using an automated COULTER cell counter (Beckman Coulter, Brea, CA, USA). BAL cell differential was determined by cytospins using Diff-Quik (Dade Behring AG, Düdingen, Switzerland) cytological staining. At least 100 cells/high-power field were counted, and a total of three fields were counted for each BAL specimen. Total protein was quantified using the bicinchoninic acid protein assay (Thermo Fisher; product #23225). LDH was measured by a Cytotoxicity Detection Kit (Roche, Basel, Switzerland; item #11644793001).

### 2.4. Histology

Mice were anesthetized, the pulmonary intravascular space was cleared, and whole lungs were dissected from mice and immediately placed in 4°C buffered formalin overnight for paraffin embedding [44]. Histological analysis was performed on 5 *μ*m sections by Yale Histopathology Core. Hematoxylin and eosin (H&E) staining was used to assess structural integrity and presence or absence of inflammation.

### 2.5. Cell Death and Apoptosis Assays

For overall cell death, lactate dehydrogenase (LDH) was measured in cell culture supernatants and cellular lysates using the Cytotoxicity Detection Kit (Roche; item #11644793001). TUNEL staining was completed on mouse tissue per the manufacturer's protocol (Roche Molecular Biochemicals, Indianapolis, IN, USA; item #11767291910) by the Yale Histopathology Services. Cells with brown nuclei were determined to be positive. For each sample, the number of TUNEL-positive alveolar epithelial cells was expressed as percentage of positive cells in area of highest positive labeling (“hot spot”) out of the total alveolar epithelial cells, and 3 hot spots were counted in each case under high-power field (400x).

### 2.6. Immunofluorescence TUNEL Assay with Immunohistochemistry Costaining

Formalin-fixed, paraffin-embedded mouse lung was labeled with rabbit anti-von Willebrand factor (VWF) (Bioss, Woburn, MA, USA; catalog # bs-100048r) or rabbit Anti-Prosurfactant Protein C (SPC) (EMD Millipore Corp., Burlington, MA, USA; catalog #AB3786) antibodies, unconjugated 1 : 100, overnight at 4°C. Subsequently, the lung was incubated in secondary donkey anti-rabbit IgG, Alexa Fluor™ 546 conjugated antibody (Invitrogen, Waltham, MA, USA; catalog #A10040) at 1 : 200 dilution for 1 hour at room temperature. For detection of TUNEL-positive cells after antibody labeling, lung sections were stained using In Situ Cell Death Detection Kit, Fluorescein (Sigma-Aldrich; catalog #11684795910) according to the manufacturer's protocol. Antifade Mounting Medium with DAPI (Vector Laboratories, Newark, CA, USA; item #H-1200) was used for nucleus staining and coverslip mounting. Fluorescent images were captured using a ZEISS Axio Observer multimodal imaging microscope (ZEISS, White Plains, NY, USA) and analyzed with ZEN Microscopy Software (version 2.1, ZEISS). For fluorescence intensity quantification, 3 WT and 3 EGFR^Wa5/+^ mice were analyzed for both normoxia and hyperoxia conditions. For each mouse, 2 randomly selected high-power fields (HPFs) were analyzed.

### 2.7. Western Blotting and ELISA

Protein levels were measured using Western blot analysis [49]. For whole lung homogenates, the lung tissue was homogenized in RIPA buffer (Thermo Fisher; catalog #89900) and supplemented with protease (Roche; reference #11836170001) and the phosphatase inhibitor sodium orthovanadate (New England Biolabs (NEB), Ipswich, MA, USA; item #P0758) and sodium fluoride (NEB; item #P0759). For cell culture experiments, cellular lysates from were collected in RIPA buffer supplemented with protease and phosphatase inhibitors. Western blotting was performed using an SDS-PAGE system (Bio-Rad, Hercules, CA, USA; item #8658004). Samples were electrophoresed in a 4-20% Tris gel (Bio-Rad; item #4568095) in Tris running buffer, transferred to a PVDF membrane (Bio-Rad; item #1704157), and probed with anti-EGFR (p- and total), anti- ERK1/2 (p- and total), anti-JNK1/2 (p- and total), anti-p38 (p- and total), anti-AKT (p- and total-), anti-HIF1*α*, anti-PARP (cleaved- and total-), and anti-caspase 3 (cleaved- and total-) primary antibodies (Cell Signaling Technologies, Danvers, MA, USA). Protein band densitometry analysis was completed using ImageJ software.

### 2.8. Mitochondrial ROS Analysis

Hydrogen peroxide was used to induce mitochondrial ROS [50]. Cells were cultured in confluent cultures on a 96-well black-bottom opaque plate 24 hours prior to hydrogen peroxide application. Mitochondrial superoxide was measured using the MitoSOX™ Red mitochondrial superoxide indicator kit (Molecular Probes, Inc., Eugene, OR, USA; catalog #M36008). MitoSOX™ Red was added to cells per the manufacturer's protocol, MitoSOX™ Red was subsequently aspirated off cells, cells were washed twice with phosphate-buffered saline (PBS), and hydrogen peroxide (5 mM; Millipore, Burlington, MA, USA; item# 386790) was added to cells with or without gefitinib. Relative fluorescence was measured every 5 minutes at 37°C on a Cytation™ 3 Automated Fluorescence Microscopy reader (BioTek, Winooski, VT, USA).

### 2.9. Single-Cell RNA Sequencing Analysis

The GSE128944 [51] and GSE132901 [52] datasets were downloaded from the Gene Expression Omnibus, a publicly available genomics data repository (available at https://www.ncbi.nlm.nih.gov/geo/). Analysis of single-cell RNA sequencing (scRNA seq) data has been described [51]. Briefly, the R package Seurat (version 4) was used to perform scRNA seq analysis. The CreateSeuratObject() was used to create the object by using default settings. Data was subsequently normalized using the logNormalize method for all the Seurat objects. Principal component analysis (PCA) was used to reduce the dataset into a smaller number of components (eigengenes) while preserving the variation of the entire data set. SingleR and Celldex R packages were used to annotate the cell types. VlnPlot() was used to create the graph for EGFR expression level among the cell types.

### 2.10. Statistical Analysis

Molecular data were examined by analyses of variance to estimate means and standard errors. Statistical significance was assessed by two-tailed *t*-tests, Mann–Whitney tests, and analysis of variance. Survival data were examined by constructing Kaplan-Meier plots, and statistical significance was assessed by log-rank chi-square tests. Graphs and statistical analyses were completed using GraphPad Prism version 9.2.0 (GraphPad Software version 9.2.0, La Jolla, CA, USA).

## 3. Results

### 3.1. EGFR Inhibition Is Protective in Hyperoxia-Induced Lung Injury *In Vivo*

**Figure 1:**
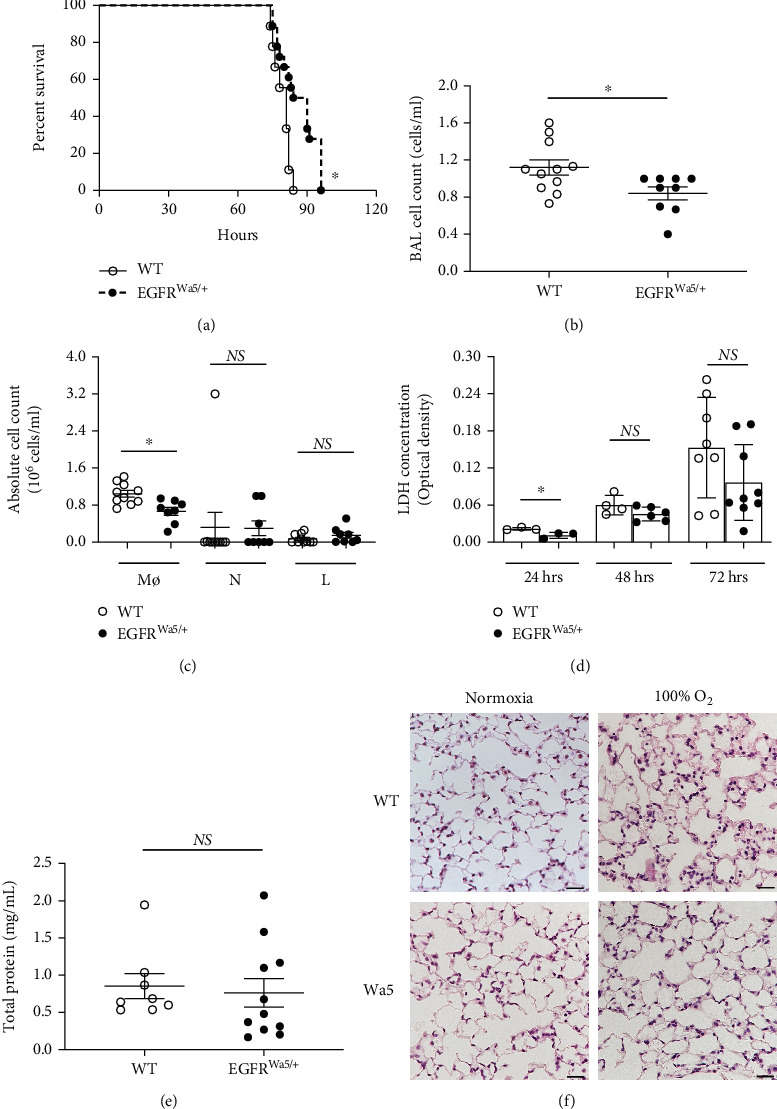
EGFR inhibition improves survival and reduces acute lung injury in hyperoxia. (a) Effects of severe hyperoxia (100% oxygen) on survival were examined in EGFR^Wa5/+^ mice vs. WT littermates (*N* = 5-6 mice/group, repeated twice) ∗*p* < 0.05, log-rank analysis. (b–f) BAL and lungs were analyzed in EGFR^Wa5/+^ and WT mice subjected to hyperoxia for 24 h (*N* = 5-6 mice/group, repeated once), 48 h (*N* = 5-6 mice/group, repeated twice), and 72 h (*N* = 5-6 mice/group, repeated twice). BAL at 48 h: (b) cell count. ∗*p* < 0.05, Mann-Whitney *U* test. (c) Cell differential. ∗*p* < 0.05, Mann-Whitney *U* test. (d) 24, 48, and 72 h for BAL lactate dehydrogenase (LDH). ∗*p* < 0.05, Mann-Whitney *U* test. (e) BAL total protein at 48 h. (f) Histopathology (H&E) of lungs from EGFR^Wa5/+^ and WT mice at 72 h severe hyperoxia (representative of *N* = 5-6 mice/group, repeated twice, scale bar = 100 *μ*m). BAL: bronchoalveolar lavage; L: lymphocytes; M*ϕ*: macrophages; N: neutrophils; Wa5: EGFR^Wa5/+^ mice; WT: wild type.

Based on the role of EGFR in cell proliferation [23, 24], wound repair [25, 26], and lung injury [21, 22], we initially hypothesized that EGFR inhibition would lead to increased susceptibility to hyperoxia-induced lung injury. Here, genetically modified Waved-5 (EGFR^Wa5/+^) mice, which have a point mutation that decreases EGFR activity significantly [41], were used. EGFR^Wa5/+^ and WT littermates were subjected to severe hyperoxia (100% oxygen), and survival was observed (Figure 1(a)). In this model, instead of being deleterious, EGFR inhibition in EGFR^Wa5/+^ mice resulted in improved survival compared with WT (Figure 1(a)). Both EGFR^Wa5/+^ and WT mice showed 100% survival until approximately 70 hours, after which survival significantly decreased in the following 20 hours, which is consistent with prior studies for the genetic background (BALB/c, C3H, and C57BL/6J) [53]. To further characterize the protective effect of EGFR inhibition in severe hyperoxia *in vivo*, lung injury was analyzed in EGFR^Wa5/+^ mice exposed to 100% oxygen. EGFR^Wa5/+^ and WT mice were subjected to severe hyperoxia for 24, 48, and 72 hours, and bronchoalveolar lavage (BAL) and lungs were collected for analysis. In these conditions, EGFR^Wa5/+^ mice showed decreased total cell count (Figure 1(b)) compared with WT. In order to determine the cell type yielding this difference, absolute cell number for each cell type was calculated, which showed that EGFR^Wa5/+^ mice contained reduced macrophages compared with WT (Figure 1(c)). In addition, EGFR^Wa5/+^ mice showed decreased BAL lactate dehydrogenase (LDH) compared with WT at 24 hours (Figure 1(d)). This trend persisted at 48 and 72 hours, although the difference was not statistically significant at these later time points (Figure 1(d)). No differences were observed in BAL fluid total protein (Figure 1(e)). EGFR^Wa5/+^ mice also showed decreased histological acute lung injury compared with WT, as evidenced by decreased inflammatory cell infiltrates (Figure 1(e)). Together, these data show that decreased EGFR activity during severe hyperoxia *in vivo* is associated with increased survival and decreased acute lung injury.

### 3.2. Endothelial-Specific EGFR Knockout Is Not Protective in Hyperoxia-Induced Lung Injury

**Figure 2:**
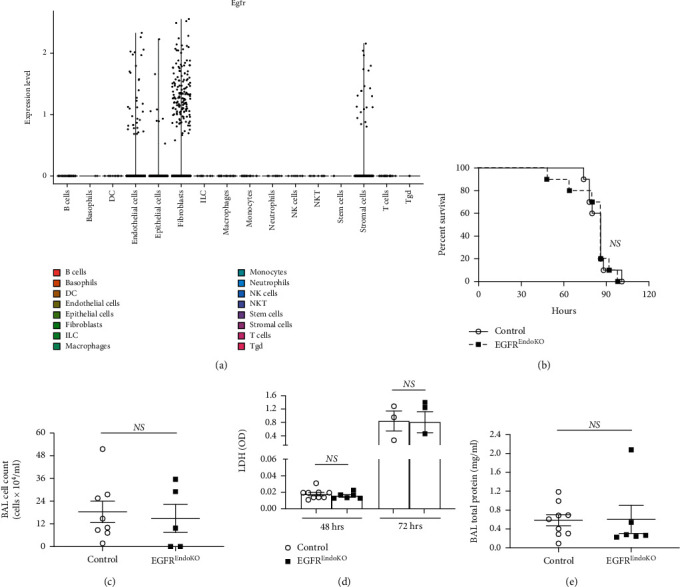
Endothelial-specific EGFR knockout is not protective in hyperoxia-induced lung injury. (a) To determine EGFR expression by pulmonary cell type, publicly available datasets of single-cell RNA sequencing (scRNA seq) on the mouse lung were analyzed as described in the methods. EGFR expression levels for each cell type is shown. (b) Effects of severe hyperoxia (100% oxygen) on survival were examined in EGFR^EndoKO^ mice vs. control littermates (*N* = 5-6 mice/group). (c–e) BAL and lungs were analyzed in EGFR^EndoKO^ mice and WT mice subjected to hyperoxia for 48 h (*N* = 5-6 mice/group) and 72 h (*N* = 5-6 mice/group). BAL at 48 h: (c) cell count. (d) 48 h and 72 h for BAL lactate dehydrogenase (LDH). (e) BAL total protein at 48 h. BAL: bronchoalveolar lavage; DC: dendritic cell; EGFR^EndoKO^: mice with endothelial-specific knockout of EGFR; ILC: innate lymphoid cell; NK cell: natural killer cell; NKT: natural killer T cell; Tgd: *γδ* T cell.

The above experiments showed that decreased EGFR activity results in improved survival and decreased lung injury in severe hyperoxia. Based on the established role of the endothelial and epithelial cell compartments in hyperoxia-induced lung injury [12], we analyzed publicly available single cell RNA sequencing data of a model of murine acute lung injury [51] and of mouse lungs [52] to determine cell-type-specific EGFR expression. This analysis showed high EGFR expression in epithelial and endothelial cells (Figure 2(a)), which has been shown previously in lung epithelial [54] and endothelial [55] cells.

Based on these results, we investigated whether endothelial-specific EGFR knockout leads to improved survival in hyperoxia. Transgenic endothelial-specific EGFR knockout mice (VE-Cadherin-Cre x EGFR^fl/fl^ (EGFR^EndoKO^)) and controls were exposed to severe hyperoxia. No differences in survival were seen (Figure 2(b)). In these experiments, control mice (genetic background C57BL/6J [42, 43]) showed median survival of 86 hours (Figure 2(b)), which was greater than median survival of WT mice in the EGFR^Wa5/+^ survival experiments (genetic background BALB/c, C3H, and C57BL6/6J [41]; WT median survival = 81 hours; Figure 1(a)), consistent with known different susceptibility patterns to hyperoxia in certain genetic mouse strains [53]. In addition, effects of 100% oxygen on acute lung injury were examined in EGFR^EndoKO^ and controls at 48 and 72 hours, and no differences were seen in BAL cell count (Figure 2(c)), BAL LDH (Figure 2(d)), and BAL total protein (Figure 2(e)).

### 3.3. Decreased Pulmonary Cell Death in EGFR^Wa5/+^ Mice with Severe Hyperoxia

**Figure 3:**
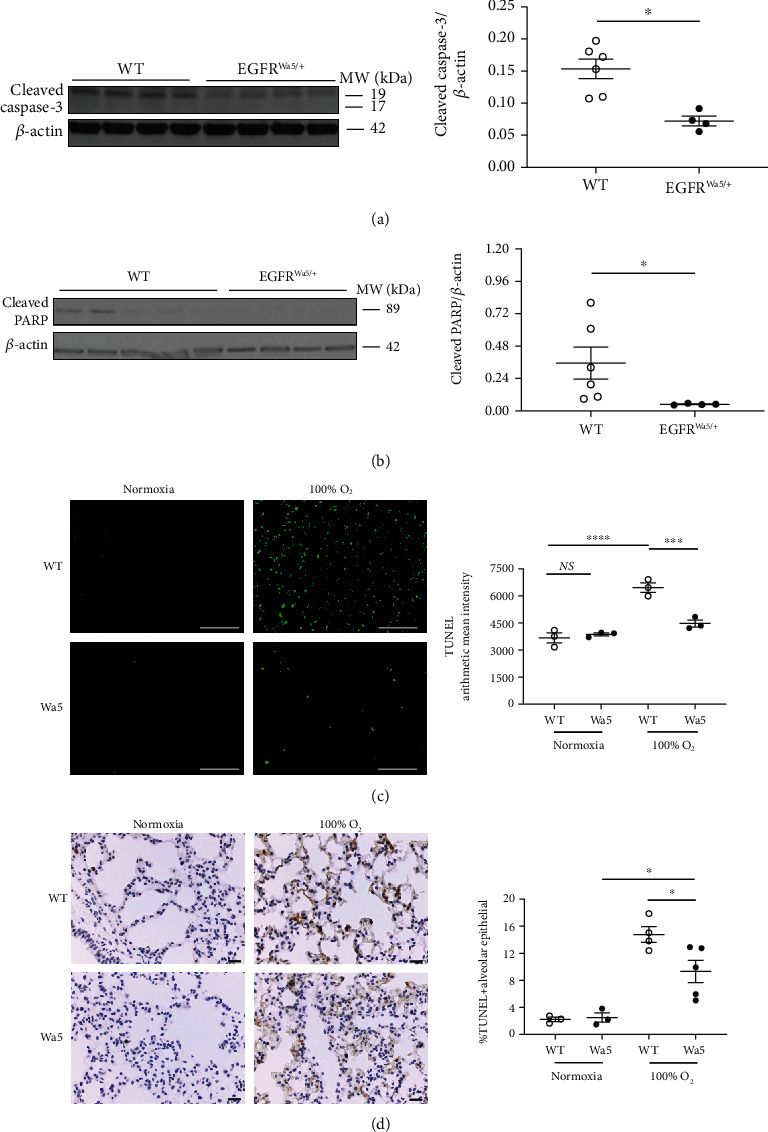
EGFR inhibition decreases pulmonary cell death and apoptotic markers in hyperoxia-induced lung injury. (a–d) Effects of severe hyperoxia (100% oxygen) were examined in EGFR^Wa5/+^ and WT mice at 24 h (*N* = 5-6 mice/group, repeated once), 48 h (*N* = 5-6 mice/group, repeated twice), and 72 h (*N* = 5-6 mice/group, repeated twice). (a) Western blot (WB) for cleaved caspase-3 at 48 h in whole lungs. Representative samples run in duplicate are shown (representative of three independent experiments). Quantitative densitometry using *β*-actin is shown. ∗*p* < 0.05, Mann-Whitney *U* test. (b) WB for cleaved PARP at 48 h in the whole lungs. WB of three independent experiments is shown. Quantitative densitometry using *β*-actin is shown. ∗*p* < 0.05, Mann-Whitney *U* test. (c, d) TUNEL on lungs from EGFR^Wa5/+^ and WT mice at 72 h severe hyperoxia (representative of three independent experiments, *N* = 5-6 mice/group, scale bar = 100 *μ*m). (c) TUNEL immunofluorescent staining. Quantification of TUNEL arithmetic mean intensity is shown. ∗∗∗*p* < 0.0005, ∗∗∗∗*p* < 0.0001, 2-way ANOVA. (d) TUNEL staining with hematoxylin counterstain. Quantification of TUNEL-positive alveolar epithelial cells expressed as percentage of total number of alveolar epithelial cells is shown. ∗*p* < 0.05, unpaired *t* test. PARP: poly (ADP-ribosyl) polymerase; TUNEL: terminal dUTP nick end labeling; Wa5: EGFR^Wa5/+^ mice; WT: wild type.

Severe hyperoxia induces epithelial and endothelial cell death [12], and thus, the potential for decreased EGFR to modulate cell death in 100% oxygen was investigated in EGFR^Wa5/+^ and WT mice (Figure 3). Markers of apoptosis included caspase-3, which is an effector caspase responsible for the characteristic morphological changes of apoptosis, including membrane blebbing, cell shrinkage, formation of “apoptotic bodies,” and chromosomal DNA fragmentation [56]. Activated (i.e., cleaved) caspase-3 cleaves caspase substrates, including poly (ADP-ribosyl) polymerase (PARP), leading to DNA fragmentation and cell death [14]. Therefore, cleaved portions of caspase-3 and PARP were used to measure apoptosis. EGFR^Wa5/+^ mice showed decreased cleaved fractions of whole lung caspase-3 (Figure 3(a)) and PARP (Figure 3(b)) at 48 hours compared with WT by Western blot. In addition, cell death was examined in lungs of EGFR^Wa5/+^ and WT mice exposed to 100% oxygen for 72 hours by terminal dUTP nick end labeling (TUNEL) immunofluorescence staining. No differences between EGFR^Wa5/+^ and WT were observed in normoxia (Figure 3(c)). Hyperoxia significantly increased pulmonary cell death in WT compared with normoxia, and with hyperoxia EGFR^Wa5/+^ mice showed reduced TUNEL intensity compared with WT mice (Figure 3(c)). To investigate the cell type involved in this difference, costaining was used to identify TUNEL-positive alveolar and endothelial cells. EGFR^Wa5/+^ mice showed reduced TUNEL-positive alveolar (Supplemental Figure S1A) and endothelial cells (Supplemental Figure S.2A) compared with WT in hyperoxia. Furthermore, histological analysis of TUNEL-positive alveolar epithelial cells showed the following: (1) increased TUNEL-positive cells in both WT and EGFR^Wa5/+^ in hyperoxia compared with normoxia and (2) EGFR^Wa5/+^ mice contained decreased TUNEL+ cells compared with WT mice in hyperoxia (3D). Together, these data show that EGFR inhibition *in vivo* leads to reduced cell death in hyperoxia-induced lung injury.

### 3.4. EGFR^Wa5/+^ Mice Show Reduced Pulmonary EGFR and ERK Activation in Hyperoxia

**Figure 4:**
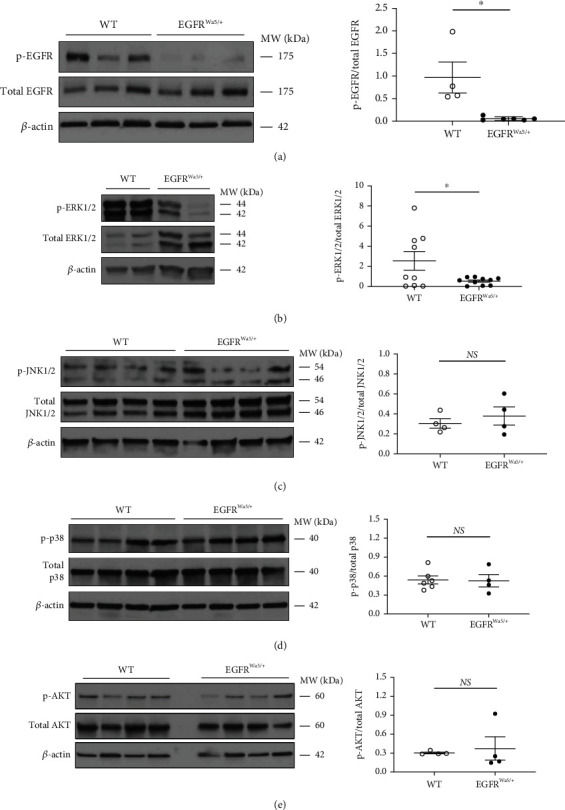
EGFR is activated in hyperoxia and modulates ERK, but not JNK, p38, and AKT *in vivo*. Effects of severe hyperoxia (100% oxygen) were examined in lungs from EGFR^Wa5/+^ and WT mice at 24 (*N* = 5-6 mice/group, repeated once), 48 (*N* = 5-6 mice/group, repeated twice), and 72 h (*N* = 5-6 mice/group, repeated twice). (a) Western blot (WB) for EGFR (p- and total) at 48 h. ∗*p* < 0.05, Mann-Whitney *U* test. (b) WB for ERK1/2 (p- and total) at 24 h. ∗*p* < 0.05, unpaired *t* test. (c) WB for JNK1/2 (p- and total) at 48 h. (d) WB for p38 (p- and total) at 48 h. (e) WB for p-AKT (p- and total) at 72 h. WB of two independent experiments (24 h) and three independent experiments (48 h and 72 h) is shown. Densitometry using total fraction is shown. ERK1/2: extracellular signal-regulated kinase 1 and 2; JNK1/2: c-Jun N-terminal kinase 1 and 2; p38: protein 38; WT: wild type.

Based on improved survival and reduced cell death in EGFR^Wa5/+^ compared with WT mice in 100% oxygen (Figures 1 and 3), we investigated pathways associated with cell survival/death in severe hyperoxia. First, whole-lung EGFR activity was measured in EGFR^Wa5/+^ and WT mice subjected to 100% oxygen for 48 hours. Whole lung EGFR phosphorylation (p-EGFR) was evaluated by Western blot (Figure 4(a)). As anticipated, p-EGFR was decreased in EGFR^Wa5/+^ mice compared to WT (Figure 4(a)). Based on the role of MAP kinases in hyperoxia-induced lung injury [12, 13] and that EGFR is upstream from ERK [16, 20, 57], JNK [58], and p38 [59], pulmonary expression of each of these MAP kinases was investigated in hyperoxia-induced lung injury in EGFR^Wa5/+^ and WT mice by Western blot (Figures 4(b)–4(d)). In EGFR^Wa5/+^ versus WT mice, there was no difference in p-ERK1/2 in normoxia at 24 hours (data not shown). However, in severe hyperoxia at 24 hours, EGFR^Wa5/+^ mice showed significantly decreased p-ERK1/2 compared with WT (Figure 4(b)). In addition, no differences were observed for p-JNK (Figure 4(c)) or p-p38 (Figure 4(d)). AKT also regulates cell survival and is modulated by EGFR in severe hyperoxia [16]. Thus, whole lung AKT activity was also investigated, and no difference in p-AKT was observed (Figure 4(e)). Finally, hyperoxia is known to induce hypoxia-inducible factor-1*α* (HIF-1*α*) [60], and HIF-1*α* is known to regulate apoptosis dependent on oxygen concentrations [61]. Thus, whole lung HIF-1*α* levels were investigated in severe hyperoxia at 48 hours, and no difference in HIF-1*α* was identified between EGFR^Wa5/+^ and WT mice (data not shown).

### 3.5. EGFR Inhibition in Hyperoxia Decreases ERK Activation and Cell Death in Alveolar Epithelial Cells

**Figure 5:**
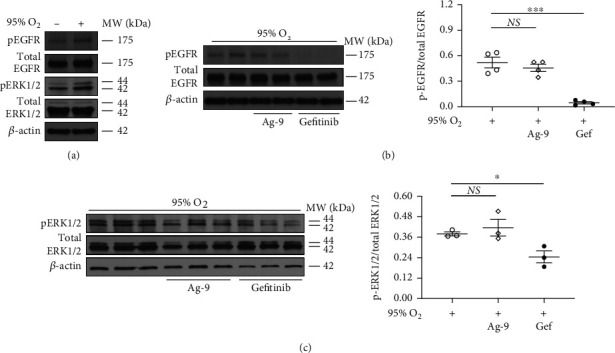
Hyperoxia activates ERK via EGFR in murine alveolar epithelial cells. Effects of severe hyperoxia (95% oxygen) were examined in MLE12 cells treated with EGFR-selective tyrosine kinase inhibitor gefitinib and compared with normoxia (repeated twice). Ag-9 was used as a negative control. Western blot (WB) for (a) EGFR (p- and total) and ERK1/2 (p- and total) at 60 minutes. Representative sample is shown (representative of three independent experiments). (b) EGFR (p- and total) at 60 minutes. WB of three independent experiments is shown. Quantitative densitometry of 2 representative samples run in duplicate using total fraction is shown. ∗∗∗*p* < 0.0005, unpaired *t* test. (c) ERK1/2 (p- and total) at 48 h. Quantitative densitometry using total fraction is shown. ∗*p* < 0.05, unpaired *t* test. ERK1/2: extracellular signal-regulated kinase 1 and 2; Gef: gefitinib; Hyp: hyperoxia; MLE12: murine lung epithelial 12.

Based on the single-cell RNA sequencing analysis in acute lung injury that showed high epithelial EGFR expression (Figure 2(a)), the known high expression of EGFR in lung injury [24, 54], and reduced alveolar epithelial cell death in EGFR^Wa5/+^ mice compared with WT (Figure 3(d)), we next investigated the role of EGFR inhibition in alveolar epithelium *in vitro*. First, effects of severe hyperoxia (95% oxygen) on p-EGFR and MAP kinases ERK, JNK, and p38 activation were examined via Western blot in MLE12 cells, a mouse alveolar epithelial cell line, compared with normoxia controls. Severe hyperoxia activated EGFR (Figure 5(a)) and ERK (Figure 5(a)) at 60 minutes, which is consistent with the kinetics of EGFR activation by other stimuli in lung epithelial cells [33]. Treatment with the EGFR-selective tyrosine kinase inhibitor gefitinib (Iressa®) reduced EGFR activation (Figure 5(b)), which was not seen with an inactive analog (Ag-9) that serves as control for EGFR tyrosine kinase inhibition [62]. In addition, gefitinib, but not Ag-9, reduced ERK activation at 48 hours (Figure 5(c)). However, JNK and p38 activation was not affected by gefitinib (data not shown).

**Figure 6:**
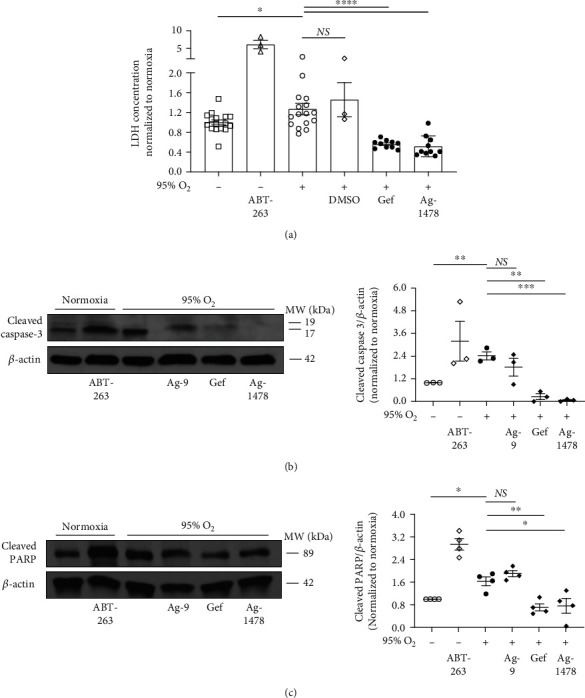
EGFR inhibition decreases cell death and apoptotic markers in murine alveolar epithelial cells in severe hyperoxia. Effects of severe hyperoxia (95% oxygen) were examined in MLE12 cells treated with EGFR-selective tyrosine kinase inhibitors gefitinib and Ag-1478 and compared with normoxia controls (repeated twice). ABT-263, an apoptosis inducer, was used as positive control. DMSO and Ag-9 were used as negative controls. (a) Lactate dehydrogenase (LDH) at 72 h. Representative of three independent experiments. LDH concentration values for each experiment were normalized to the average normoxia LDH concentration for that experiment. ∗*p* < 0.05, ∗∗∗∗*p* < 0.001 unpaired *t* test. (b, c) Western blot (WB) for (b) cleaved caspase-3 and (c) cleaved PARP at 48 h. WB of three independent experiments is shown. Quantitative densitometry using *β*-actin is shown. Quantitative densitometry values for each WB were normalized to the normoxia densitometry value (i.e., control value) for that WB. ∗*p* < 0.05, ∗∗*p* < 0.005, and ∗∗∗*p* < 0.0005; unpaired *t* test. Gef: gefitinib; Hyp: hyperoxia; PARP: poly (ADP-ribosyl) polymerase.

Next, because results showed decreased alveolar cell death in mice with reduced EGFR activity (Figure 3(d)), we examined the effects of EGFR inhibition on cell death in severe hyperoxia in MLE12 cells. MLE12 cells were subjected to 95% oxygen or normoxia and treated with or without EGFR tyrosine kinase inhibitors (gefitinib or Ag-1478), and markers of apoptosis were measured. ABT-263, an apoptosis inducer, was used as a positive control. DMSO (vehicle) and Ag-9 were used as negative controls. LDH, a marker of cell death, was measured in cell culture supernatants. Proteins identifying apoptosis (e.g., cleaved fractions of caspase-3 and PARP [56]) were evaluated by Western blot. Cell death was increased in hyperoxia compared with normoxia at 72 hours as measured by LDH (Figure 6(a)), and EGFR inhibition reduced cell death in hyperoxia (Figure 6(a)). In addition, cleaved fractions of caspase-3 (Figure 6(b)) and PARP (Figure 6(c)) were increased in hyperoxia compared with normoxia at 48 hours. EGFR inhibition with hyperoxia resulted in decreased cleaved fractions of caspase-3 (Figure 6(b)) and PARP (Figure 6(c)).

Because hyperoxia induces production of ROS which leads to cell death [12] and antioxidant mechanisms provide protection against cell death in hyperoxia [63], we next examined if EGFR inhibition affects antioxidant reserves, which would provide protection in hyperoxia. Effects of EGFR inhibition with gefitinib on mitochondrial ROS production were measured in MLE12 cells treated with hydrogen peroxide. Hydrogen peroxide induces oxidative damage via generation of mitochondrial superoxide [50], which also occurs in hyperoxia [64]. This model was used to measure mitochondrial ROS every 5 minutes for 4 hours, which is not possible in our *in vitro* hyperoxia system. Hydrogen peroxide increased mitochondrial superoxide production compared with controls (data not shown), and gefitinib treatment (1 *μ*M and 10 *μ*M) did not reduce mitochondrial superoxide production by hydrogen peroxide (Supplementary Figure S3A and S3B).

### 3.6. Genetic EGFR Deletion Attenuates Hyperoxia-Mediated Apoptosis and ERK in Alveolar Epithelial Cells

**Figure 7:**
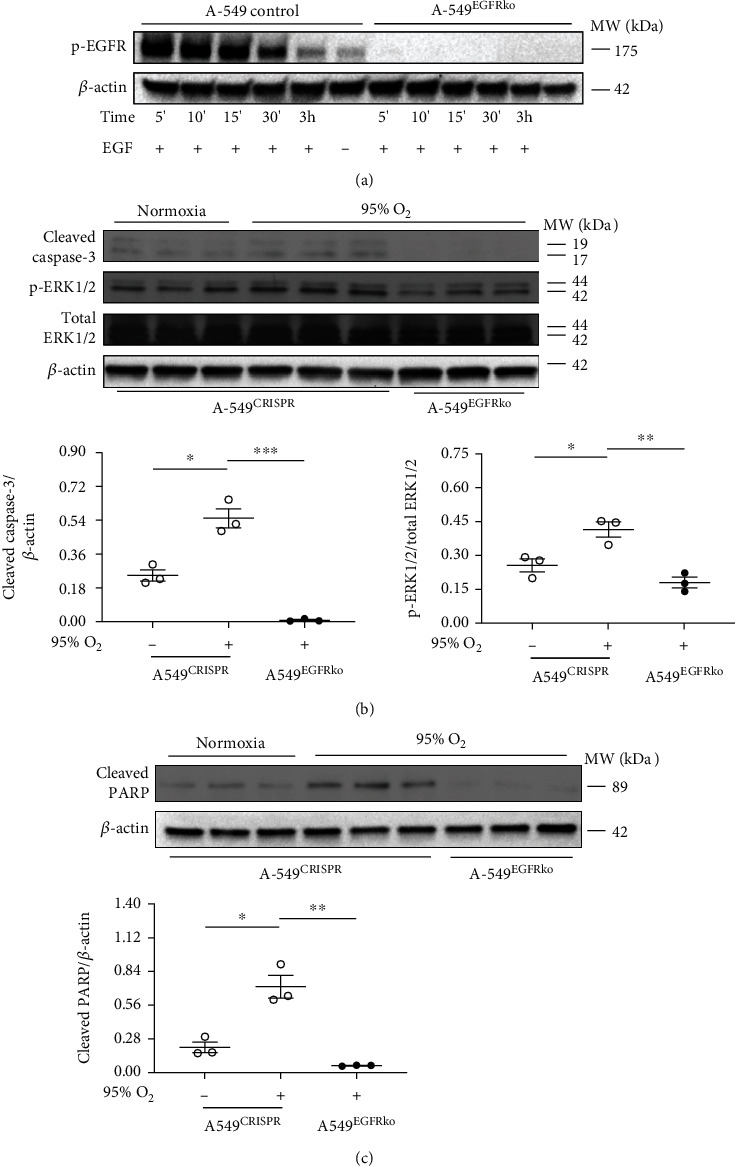
EGFR deletion via CRISPR leads to decreased apoptotic markers and reduced ERK activation in human alveolar epithelial cells in hyperoxia. (a) To confirm EGFR deletion, A-549 cells containing CRISPR-mediated EGFR deletion (A-549^EGFRko^) and relevant controls (A-549 (control)) were stimulated with EGF. Western blot (WB) for p-EGFR is shown. (b, c) Effects of severe hyperoxia (95% oxygen) were examined in A-549^EGFRko^ and relevant controls (A-549 (control) and cells containing empty CRISPR vector (A-549^CRISPR^)) and compared with normoxia. (b) WB for cleaved caspase-3 and ERK1/2 (p- and total) at 48 h. Quantitative densitometry using *β*-actin for caspase-3 and total-ERK1/2 for p-ERK is shown. ∗*p* < 0.05, ∗∗*p* < 0.005, and ∗∗∗*p* < 0.0005; unpaired *t* test. (c) WB for cleaved PARP at 48 h. Quantitative densitometry using *β*-actin is shown. ∗*p* < 0.05, ∗∗*p* < 0.005; unpaired *t* test. Hyp: hyperoxia; PARP: poly (ADP-ribosyl) polymerase.

EGFR^Wa5/+^ mice have a point mutation that causes reduced EGFR activity [41], and the above *in vitro* experiments used chemical inhibitors, which may have off-target effects. Thus, additional *in vitro* experiments were designed to genetically manipulate EGFR via CRISPR. EGFR was deleted by CRISPR in a human alveolar-like cell (A-549) line (A-549^EGFRko^) [47]. Relevant controls included A-549 alone (control) and cells containing empty CRISPR vector (A-549^CRISPR^). First, A-549^EGFRko^ and control cells were stimulated with an EGFR ligand (EGF) to induce p-EGFR (Figure 7(a)). A-549^EGFRko^ cells showed minimal p-EGFR compared with controls (Figure 7(a)), which confirmed reduction in EGFR activity in cells with EGFR-selective CRISPR modification.

Based on reduction in cell death and apoptotic markers shown in prior experiments (Figure 6), the effects of 95% oxygen on A-549^EGFRko^ were investigated. A-549^EGFRko^ and control cells were exposed to normoxia or 95% oxygen for >24 hours, and cleaved fractions of caspase-3 and PARP in cell lysates were measured. A-549^CRISPR^ cells showed increased cleaved fractions of caspase-3 (Figure 7(b)) and PARP (Figure 7(c)) in hyperoxia compared with normoxia, and A-549^EGFRko^ cells showed reduced cleaved fractions of caspase-3 (Figure 7(b)) and PARP (Figure 7(c)) compared with A-549^CRISPR^ cells in hyperoxia.

Because EGFR^Wa5/+^ mice have decreased pulmonary ERK activation compared to WT in hyperoxia (Figure 4(b)), the effects of genetic EGFR inhibition (A-549^EGFRko^ cells) on ERK in hyperoxia were studied. In A-549^CRISPR^ cells, ERK was activated in hyperoxia compared with normoxia (Figure 7(b)). A-549^EGFRko^ cells showed decreased ERK activation compared with A-549^CRISPR^ in hyperoxia (Figure 7(b)). Thus, genetic deletion of EGFR via CRISPR reduced cell death and ERK activation in hyperoxia at 48 hours. Because HIF-1*α* can be induced by hyperoxia and is known to regulate cell death, effects of CRISPR-mediated EGFR deletion on HIF-1*α* in hyperoxia were investigated. No significant difference was observed in HIF-1*α* levels in A-549^EGFRko^ cells compared to A-549^CRISPR^ cells at 48 hours (Supplemental Figure S4A).

In summary, *in vivo* genetic EGFR inhibition improved survival, decreased acute lung injury, lowered pulmonary cell death, and attenuated ERK activation in the lungs with severe hyperoxia (100% oxygen). *In vitro* in alveolar epithelial cells, hyperoxia activated EGFR and ERK, and EGFR inhibition with EGFR-selective tyrosine kinase inhibitors decreased cell death and markers of apoptosis in severe hyperoxia (95% oxygen). Genetic EGFR deletion *in vitro* via CRISPR in a human alveolar epithelial cell line resulted in decreased hyperoxia-induced apoptosis and decreased ERK activation.

## 4. Discussion

While the harm of exposure to very high oxygen levels was identified over 200 years ago [2] and the negative impact of severe hyperoxia in the lungs has been well-characterized [3–5], evidence linking severe hyperoxia with poor clinical outcomes has renewed attention on pulmonary oxygen toxicity [7, 8, 65]. While administration of oxygen is a lifesaving treatment for hypoxic respiratory failure and ARDS, unnecessarily high oxygen administration is not uncommon in intensive care units [66]. The clinical importance of oxygen toxicity prompted these studies to investigate the role of EGFR, a pleiotropic tyrosine kinase receptor that affects diverse cellular pathways from mitogenesis to apoptosis, cell migration, and wound repair [20], in hyperoxia-induced lung injury.

In other forms of acute lung injury, EGFR activation can have both beneficial and deleterious effects in the lung. Thus, a novel finding in this study is that in severe hyperoxia (100% oxygen) EGFR inhibition is protective for mice and reduces cell death both *in vivo* and *in vitro*. Favorable effects of EGFR activation include restoration of epithelial cell barrier function after injury [28], wound closure, cell proliferation, and cell migration in alveolar epithelial cells [23, 26, 27], and lung repair [21, 22, 67]. However, EGFR activation has been implicated in alveolar epithelial processes that drive lung injury; specifically in models of lung injury caused by mechanical stretch (i.e., VILI) [29], pulmonary fibrosis induced by overexpression of transforming growth factor-*α* (TGF-*α*), an EGFR ligand [35], bleomycin-induced pulmonary fibrosis [36], and LPS-induced acute lung injury [30]. Given its diverse effects in the lung, it is likely that EGFR inhibition has both positive and negative effects in severe hyperoxia. However, the sum of the effects led to a survival benefit in this *in vivo* model. EGFR pleiotropy also helps to explain the small, but significant, *in vivo* survival curve. Thus, this study further characterizes the complex role of EGFR in acute lung injury, which may also translate to EGFR-dependent signaling in other lung diseases.

EGFR activates multiple downstream signaling pathways that lead to diverse cell responses, including MAPK and PI3K/AKT pathways [20]. ERK activation has been shown to exert both positive (i.e., prosurvival) and negative (i.e., promoting cell death) effects in epithelial cells in hyperoxia. In our study, EGFR^Wa5/+^ mice showed reduced pulmonary ERK activation in severe hyperoxia, and CRISPR-mediated EGFR deletion in alveolar epithelial cells led to decreased apoptosis and ERK activation. This finding diverges from a previously-defined ERK-mediated antioxidant cellular response in the alveolar epithelium [16] and prosurvival mechanisms in severe hyperoxia [15, 68, 69] but does correlate with other studies supporting a proapoptotic role for ERK [14, 70]. ERK activation affects diverse downstream cellular responses that may explain the observed differences in severe hyperoxia. EGFR activation induces ERK via the classically-described RAS/MAPK pathway [20]. However, in severe hyperoxia additional mechanisms of ERK activation, via increased ROS generation, have been described, which include upregulation of mitochondrial aldehyde dehydrogenase (ALDH2) [15] and NADPH oxidase 1 (NOX1) [71]. Because different mechanisms of ERK activation may exist simultaneously in severe hyperoxia, inhibition of one mechanism may induce a distinct downstream biological effect compared with inhibition of a separate mechanism that produces an opposite outcome [72]. Another possibility is that the temporal dynamics of ERK signaling affect the ultimate cellular response(s) [24, 73]. For example, in severe hyperoxia, constant ERK activation may lead to cell death compared to early activation, which has well-described protective effects in alveolar epithelium [15, 16]. This signaling paradigm aligns with the concept that the overall effect of EGFR activation in acute lung injury (e.g., beneficial or deleterious) depends on the degree of receptor activation (e.g., controlled versus sustained) and timing of activation in the injury process [24], and dissecting these intricate signals is an area that remains to be explored. For example, activation of the protein apoptosis signal-regulated kinase-1 (ASK1), which leads to cell death via both p38 and JNK, has been linked to lung injury and apoptosis in severe hyperoxia [74]. Thus, ASK1, or similar molecules, may contribute to an improved understanding of EGFR-dependent MAPK signaling in severe hyperoxia.

The context of EGFR activation also influences downstream signaling. For example, specific phosphotyrosine (Tyr(P)) activation predicts downstream intracellular signaling mechanisms [75]. Following EGFR phosphorylation, activated Tyr(P) 1068 and 1086 residues bind directly to growth factor receptor-bound protein 2 (GRB2), which leads to downstream ERK activation [75]. However, other Tyr(P) residues, including tyrosine 1148 (Y1148) and Y1173, are known to recruit Src homology and collagen (SHC) preferentially through Tyr(P) domains, as well as through Src homology 2 (SH2) domains. In both *in vivo* and *in vitro* experiments, severe hyperoxia activated EGFR Y1068. However, Y1086 may also be involved (data not shown). Thus, the timing, specific phosphotyrosines, and the potential for phosphatases to modulate these effects may lead to different EGFR-mediated outcomes.

In addition, EGFR is activated via a coordinated cell surface signaling pathway [25, 76]. This pathway involves diverse stimuli which induce Duox1-mediated ROS production in lung epithelium. ROS then stimulates metalloproteinases (e.g., TNF-*α* converting enzyme (TACE)) to cleave surface bound EGFR ligands (e.g., TGF-*α* proligand) that are released to subsequently activate EGFR. This cell surface pathway has not been studied in severe hyperoxia, although diphenyleneiodonium (DPI), an ROS inhibitor, decreased alveolar epithelial cell EGFR activation in severe hyperoxia [16]. This suggests that the cell surface pathway may be involved in hyperoxia-induced EGFR activation, an area which warrants further study. The potential for severe hyperoxia to activate different metalloproteinases that release different EGFR ligands adds to the complexity of EGFR signaling and may contribute to a better understanding of the balance of beneficial and deleterious effects of EGFR activation. In the context of prior studies, our findings suggest that severe hyperoxia induces ERK via EGFR activation to affect apoptosis in alveolar epithelial cells and the overall balance between protective and harmful EGFR-mediated cellular effects favors host harm.

This study may have future clinical relevance because this work identifies an EGFR-mediated proapoptotic pathway, which may be amenable to therapeutic modulation in conditions of severe hyperoxia (>95%). Because of the beneficial effects of EGFR on cell proliferation and wound repair, complete blockade of EGFR is not a viable therapeutic strategy. Pharmacologic inhibitors of downstream targets (e.g., ERK) could inhibit EGFR-specific effects that are deleterious and, at the same time, allow for beneficial EGFR-specific functions to continue. The approach of identifying downstream therapeutic targets builds on animal studies that show that selective blockade of signaling pathways involved in apoptosis improves survival in hyperoxia-induced lung injury [14, 63]. In addition, mitogen-activated protein kinase kinase (MEK) inhibitors, which affect ERK activation, are approved for clinical use in certain cancers, and selective ERK inhibitors are being tested in clinical trials [77]. Further, clinical trials are ongoing to investigate MAPK inhibitors in acute lung injury and ARDS [78]. Unraveling distal targets associated with different aspects of EGFR signaling will help to identify novel pharmacologic strategies that modulate cell death and simultaneously preserve beneficial EGFR-related mechanisms in acute lung injury. However, a better understanding of the timing and duration for such interventions in hyperoxia-induced lung injury will be essential for such a strategy to be effective.

### 4.1. Limitations

Our study establishes a novel role for EGFR signaling in hyperoxia-induced acute lung injury, but our study has a few limitations. First, experiments examined effects of severe hyperoxia at 95-100% oxygen, and routine use of such high oxygen levels is not consistent with critical care guidelines that recommend using the lowest possible oxygen level to achieve adequate arterial saturation [79]. However, 100% oxygen is often used in the intensive care unit (ICU) and thus has high clinical relevance. When critically ill patients have a significant clinical decompensation, 100% oxygen is used to maintain adequate arterial saturation. This is not an uncommon occurrence in the ICU. 100% oxygen is commonly used during intubation, the transitions from supine to prone positioning, when suctioning, which can be repeated with high frequency on a daily basis, and for patients that are heading to extracorporeal membrane oxygenation (ECMO). In ECMO cases, 100% oxygen may be used for a protracted time (>24 hours). In particular, during the COVID-19 pandemic, patients have required 100% oxygen for significant periods of time, which has been the experience at our center and others [80]. However, effects of EGFR inhibition at lower levels of hyperoxia with major clinical relevance (e.g., 40-70%) were not addressed in this study and warrant further investigation.

Second, although endothelial-specific EGFR knockout mice were not protected in severe hyperoxia and *in vitro* experiments investigated EGFR-specific mechanisms in epithelial cells, EGFR^Wa5/+^ mice did show reduced total pulmonary cell death compared to WT in severe hyperoxia, and co-staining of EGFR^Wa5/+^ lungs revealed both reduced epithelial and endothelial cell death. Taken together, our studies support the rationale that *complete* endothelial EGFR deletion (as seen in the EGFR^EndoKO^ mice) is not protective in severe hyperoxia, but that EGFR inhibition, but not total deletion (as seen in the EGFR^Wa5/+^ mice), provides protection against overall cell death. Studies examining the effect of EGFR in the endothelium found EGFR to be of minor importance in renal and vascular function [81]. However, human epidermal growth factor receptor 2 (HER2 (aka erbB-2)) and HER4 (aka erbB-4) activation can protect cardiomyocytes against apoptosis in chemotherapy-induced cell death, hypoxic-ischemic injury, and ROS-induced injury [82–86]. EGFR is known to heterodimerize with HER proteins and subsequently become activated [20], and our findings support the need for additional studies into the role of endothelial EGFR function, as well as other HER proteins, in hyperoxia.

Third, no significant effects on hydrogen peroxide-induced mitochondrial superoxide production in alveolar cells treated with gefitinib was observed. However, as EGFR is upstream of key pathways known to induce antioxidant responses, it is possible that EGFR inhibition regulates production of additional key ROS in hyperoxia, such as superoxide anion and hydrogen peroxide, which warrants further study.

For *in vitro* experiments, it is worth noting that we defined normoxia as approximately 21% oxygen and not 5% oxygen as in other tissues and cells [87]. Tissue oxygenation and oxygen metabolism in biological tissues will vary depending on the organ [88, 89], and oxygen concentrations in the lung substantially differ from those in peripheral tissues [87]. Our *in vitro* experiments compared effects of 95% oxygen to normoxia (i.e., approximately 21% oxygen) in pulmonary cells. However, EGFR-mediated effects in hyperoxia compared to additional physiologically relevant levels of oxygen (e.g., 5%) in other tissues warrant further study.

Finally, we found no significant changes in p-AKT in EGFR^Wa5/+^ mice compared to WT mice in our model of hyperoxia-induced lung injury. Thus, the effect of EGFR on PI3K/AKT signaling in severe hyperoxia was not investigated *in vitro*. PI3K/AKT, which is activated by p-EGFR [20], in alveolar epithelial cells exposed to severe hyperoxia [16] is suggested to be both beneficial [90] and deleterious [91]. It is possible that *in vivo* measurements of whole-lung p-AKT, and not cell-type-specific p-AKT, underestimated the effect of AKT in this system. Another consideration for a lack of evidence for EGFR-induced AKT activation in severe hyperoxia is the timing of when EGFR-dependent AKT activation might occur [16]. An approach that includes cell-specific and additional time-selective assessments could be used to further explore the potential role of EGFR to affect PI3K/AKT in severe hyperoxia.

## 5. Conclusions

While judicious use of supplemental oxygen can be lifesaving and is a key component to ARDS treatment, delivery of very high oxygen (>95%) for a prolonged duration leads to worse clinical outcomes, predisposes to infection, and directly damages the lung [3–5, 7–9, 65]. This study indicates that EGFR inhibition is protective in severe hyperoxia because *in vivo* EGFR inhibition resulted in improved survival, reduced acute lung injury, and decreased pulmonary cell death in 100% oxygen for >24 hours. *In vitro* EGFR-selective tyrosine kinase inhibitors and EGFR CRISPR deletion resulted in reduced apoptosis and decreased ERK in alveolar epithelium in severe hyperoxia. Based on the known pleiotropic effects of EGFR activation, including modulation of apoptosis in other models, this protective effect of EGFR inhibition in severe hyperoxia warrants further investigation of EGFR in additional forms of acute lung injury.

## Data Availability

The GSE128944 [51] and GSE132901 [52] datasets were downloaded from the Gene Expression Omnibus, a publicly available genomics data repository (available at https://www.ncbi.nlm.nih.gov/geo/).

## Supplementary Materials

Supplementary Materials Supplemental Figure S.1. EGFR inhibition reduces alveolar epithelial cell death in hyperoxia *in vivo*. Effects of severe hyperoxia (100% oxygen) were examined in EGFR^Wa5/+^ and WT mice at 72 h (*N* = 5-6 mice/group, repeated twice). (a) TUNEL staining with immunohistochemistry (IHC) co-staining of alveolar epithelial cells using anti-surfactant protein C (SPC) antibodies on the lungs (representative of three independent experiments, scale bar = 100 *μ*m). TUNEL+ cells, green; SPC+ cells, red; DAPI+ cell nuclei, blue. Arrows, TUNEL+ and SPC+ cells. DAPI: 4′,6-diamidino-2-phenylindole; DIC: differential interference contrast; SPC: surfactant protein C; TUNEL: terminal dUTP nick end labeling; Wa5: EGFR^Wa5/+^ mice. Supplemental Figure S.2. EGFR inhibition reduces endothelial cell death in hyperoxia *in vivo*. Effects of severe hyperoxia (100% oxygen) were examined in EGFR^Wa5/+^ and WT mice at 72 h (*N* = 5-6 mice/group, repeated twice). (a) TUNEL staining with immunohistochemistry (IHC) co-staining of endothelial cells using anti-Von Willebrand Factor (VWF) antibodies on lungs (representative of three independent experiments, scale bar = 100 *μ*m). TUNEL+ cells, green; VWF+ cells, red; DAPI+ cell nuclei, blue. Arrows, TUNEL+ and VWF+ cells. DAPI, : 4′,6-diamidino-2-phenylindole; DIC: differential interference contrast; SPC: surfactant protein C; TUNEL: terminal dUTP nick end labeling; Wa5: EGFR^Wa5/+^ mice. Supplemental Figure S.3. EGFR inhibition does not affect mitochondrial superoxide production induced by hydrogen peroxide. Effects of EGFR inhibition with gefitinib at (a) 1 *μ*M and (b) 10 *μ*M on mitochondrial ROS production were measured in MLE12 cells treated with hydrogen peroxide (5 mM). Initial fluorescence intensity measured in control cells was used as baseline, and fold change of fluorescence intensity from baseline is shown. Gef: gefitinib; H_2_O_2_: hydrogen peroxide. Supplemental Figure S.4. EGFR deletion via CRISPR does not affect HIF-1*α* in hyperoxia in alveolar epithelial cells. Effects of severe hyperoxia (95% oxygen) were examined in A-549^EGFRko^ and relevant controls (A-549 (control) and cells containing empty CRISPR vector (A-549^CRISPR^)) and compared with normoxia. (a) WB for HIF-1*α* at 48 h. Quantitative densitometry using *β*-actin is shown. In addition, full representative Western blot plates with molecular weight markers are presented.
