# Supplementary material for: Epidermal Growth Factor Receptor Inhibition Is Protective in Hyperoxia-Induced Lung Injury
Source: Oxid Med Cell Longev. 2022 Sep 20;2022:9518592. doi: 10.1155/2022/9518592 (PMC9526641; doi:10.1155/2022/9518592)
Supplement: Supplementary Materials — Supplemental Figure S.1. EGFR inhibition reduces alveolar epithelial cell death in hyperoxia in vivo. Effects of severe hyperoxia (100% oxygen) were examined in EGFRWa5/+ and WT mice at 72 h (N = 5-6 mice/group, repeated twice). (a) TUNEL staining with immunohistochemistry (IHC) co-staining of alveolar epithelial cells using anti-surfactant protein C (SPC) antibodies on the lungs (representative of three independent experiments, scale bar = 100 μm). TUNEL+ cells, green; SPC+ cells, red; DAPI+ cell nuclei, blue. Arrows, TUNEL+ and SPC+ cells. DAPI: 4′,6-diamidino-2-phenylindole; DIC: differential interference contrast; SPC: surfactant protein C; TUNEL: terminal dUTP nick end labeling; Wa5: EGFRWa5/+ mice. Supplemental Figure S.2. EGFR inhibition reduces endothelial cell death in hyperoxia in vivo. Effects of severe hyperoxia (100% oxygen) were examined in EGFRWa5/+ and WT mice at 72 h (N = 5-6 mice/group, repeated twice). (a) TUNEL staining with immunohistochemistry (IHC) co-staining of endothelial cells using anti-Von Willebrand Factor (VWF) antibodies on lungs (representative of three independent experiments, scale bar = 100 μm). TUNEL+ cells, green; VWF+ cells, red; DAPI+ cell nuclei, blue. Arrows, TUNEL+ and VWF+ cells. DAPI, : 4′,6-diamidino-2-phenylindole; DIC: differential interference contrast; SPC: surfactant protein C; TUNEL: terminal dUTP nick end labeling; Wa5: EGFRWa5/+ mice. Supplemental Figure S.3. EGFR inhibition does not affect mitochondrial superoxide production induced by hydrogen peroxide. Effects of EGFR inhibition with gefitinib at (a) 1 μM and (b) 10 μM on mitochondrial ROS production were measured in MLE12 cells treated with hydrogen peroxide (5 mM). Initial fluorescence intensity measured in control cells was used as baseline, and fold change of fluorescence intensity from baseline is shown. Gef: gefitinib; H2O2: hydrogen peroxide. Supplemental Figure S.4. EGFR deletion via CRISPR does not affect HIF-1α in hyperoxia in alveolar epithelial cel [file 9518592.f1.pdf]

S.1A

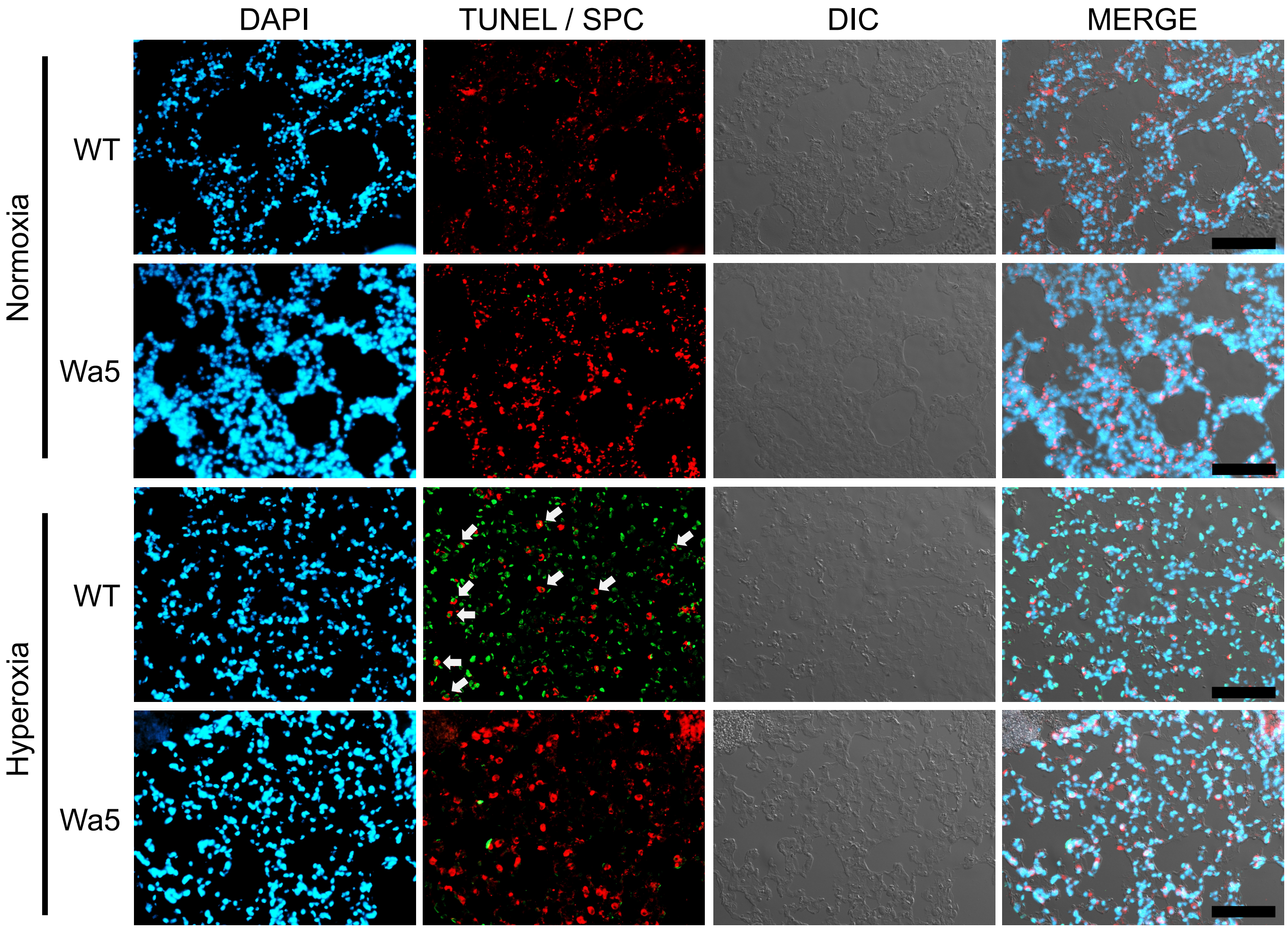

S.2A

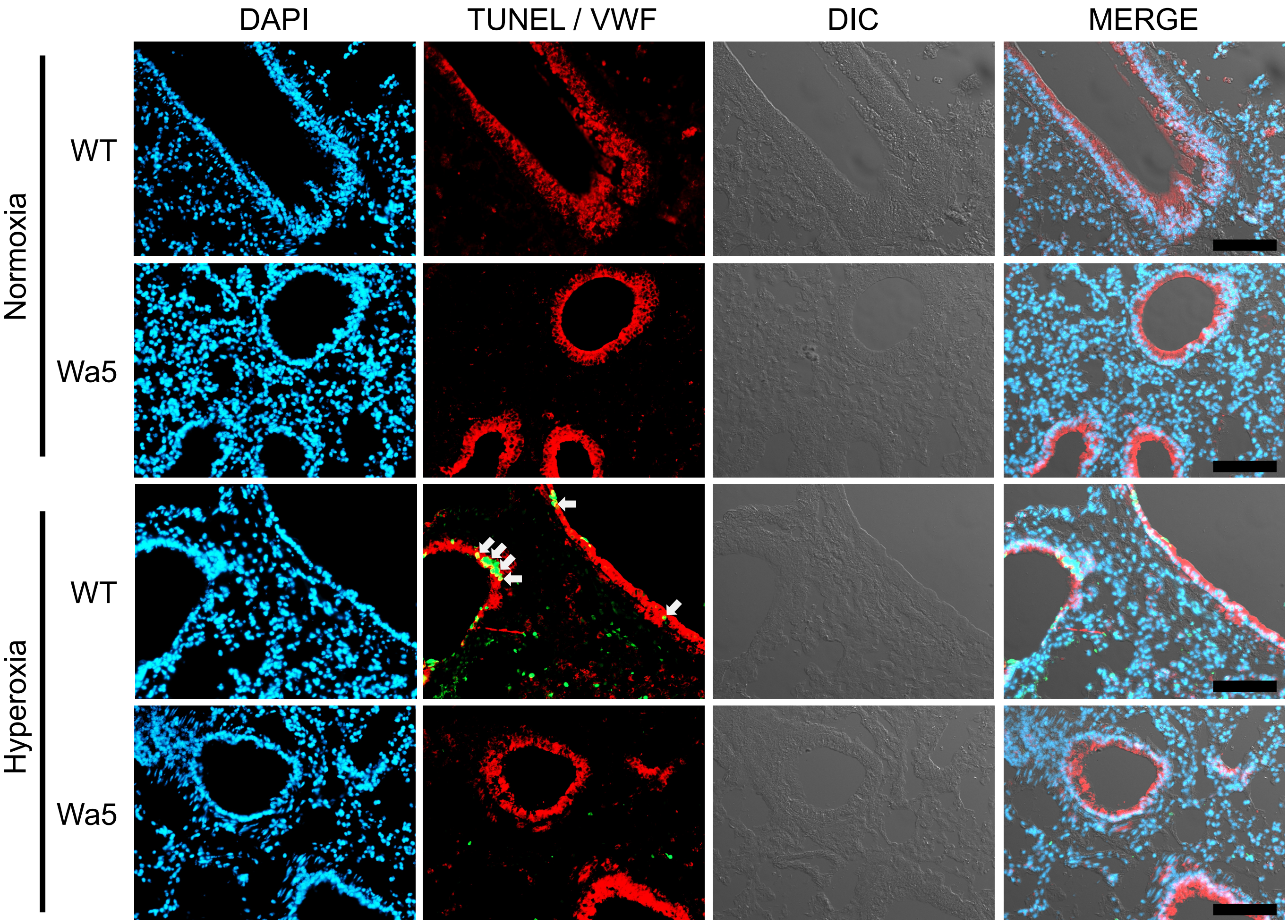

S.3A

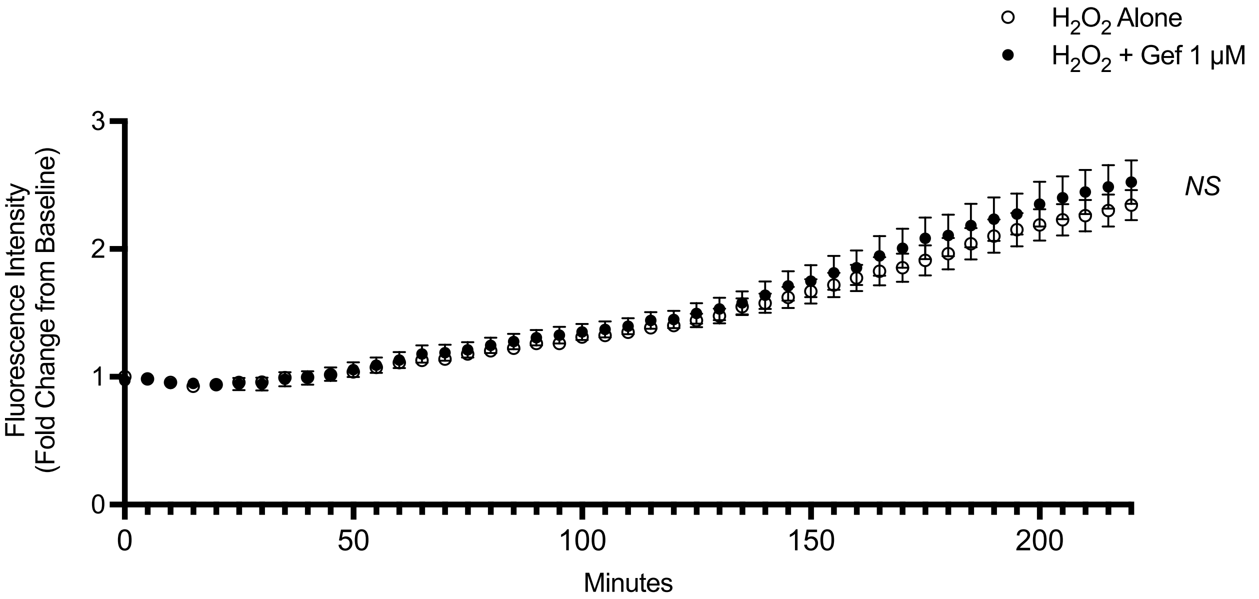

H2O2 Alone
H2O2 + Gef 1 µM
Fluorescence Intensity (Fold Change from Baseline)
Minutes
NS
0
1
2
3
0
50
100
150
200

S.3B

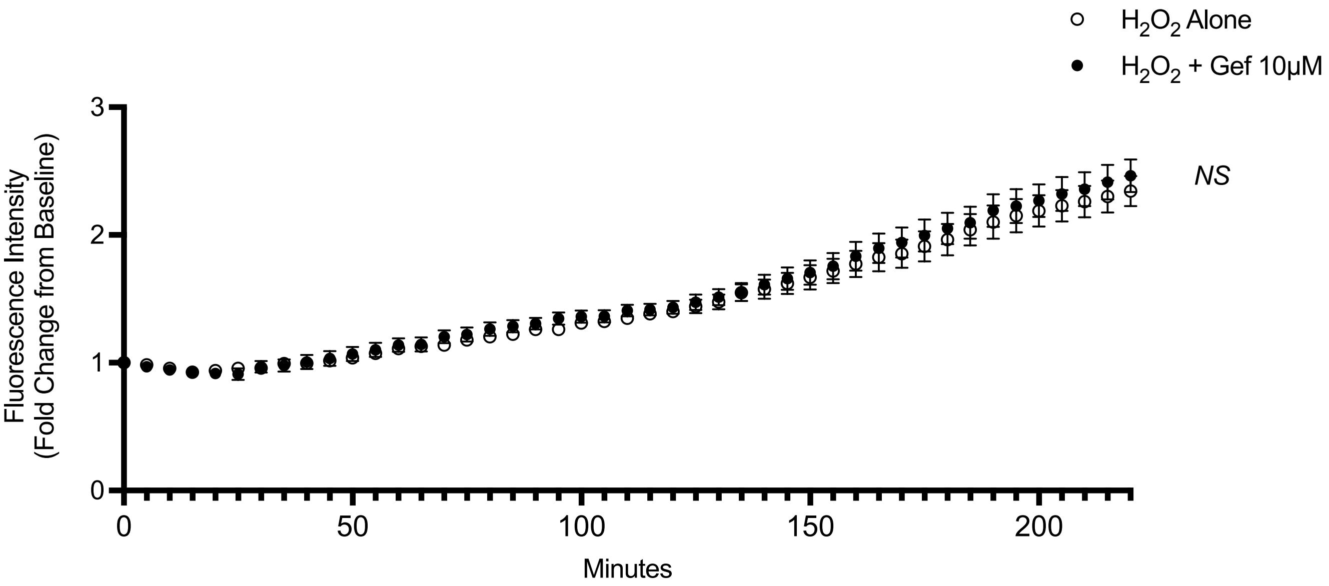

H2O2 Alone
H2O2 + Gef 10µM
Fluorescence Intensity (Fold Change from Baseline)
Minutes
NS
0
1
2
3
0
50
100
150
200

S.4A

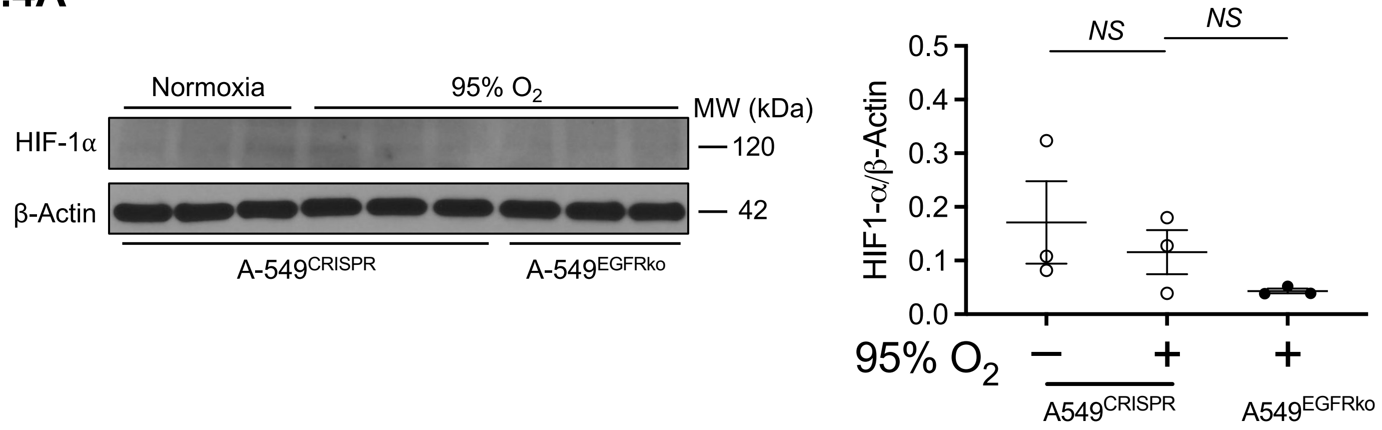
Normoxia
95% O2
MW (kDa)
HIF-1α
120
β-Actin
42
A-549CRISPR
A-549EGFRko
NS
NS
HIF1-α/β-Actin
0.5
0.4
0.3
0.2
0.1
0.0
95% O2
−
+
+
A549CRISPR
A549EGFRko

Western Blot Plates

WBs for Figure 3A

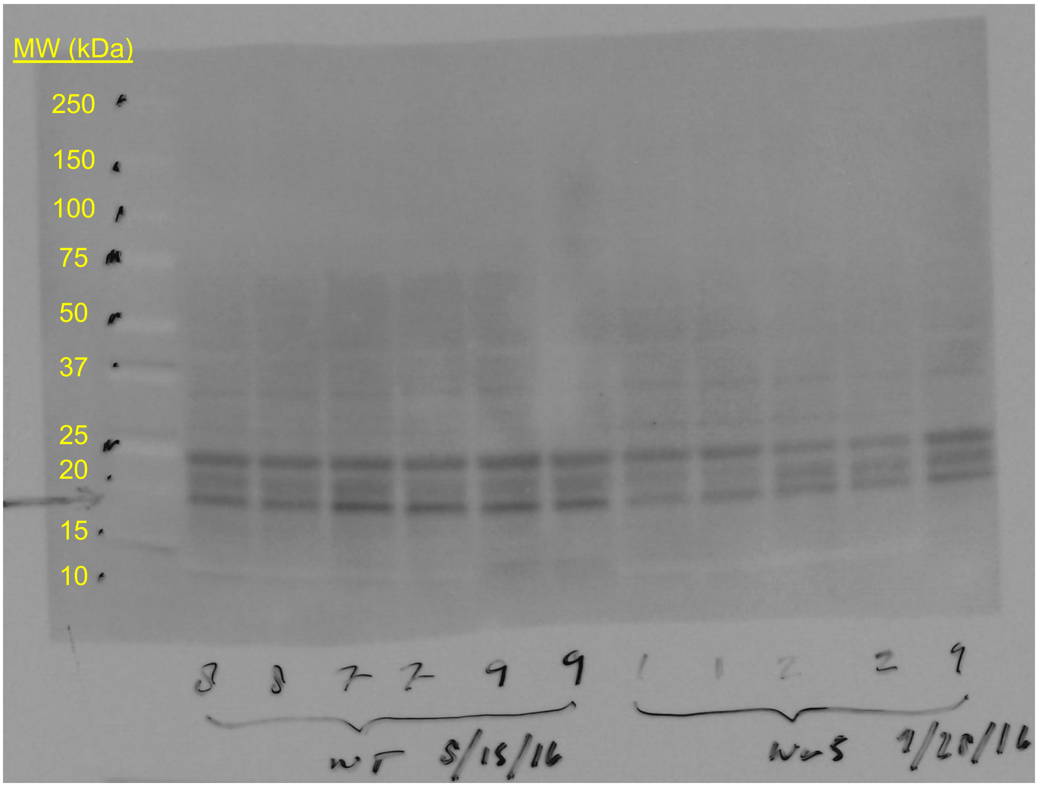

Cleaved caspase-3 (17, 19 kDa)

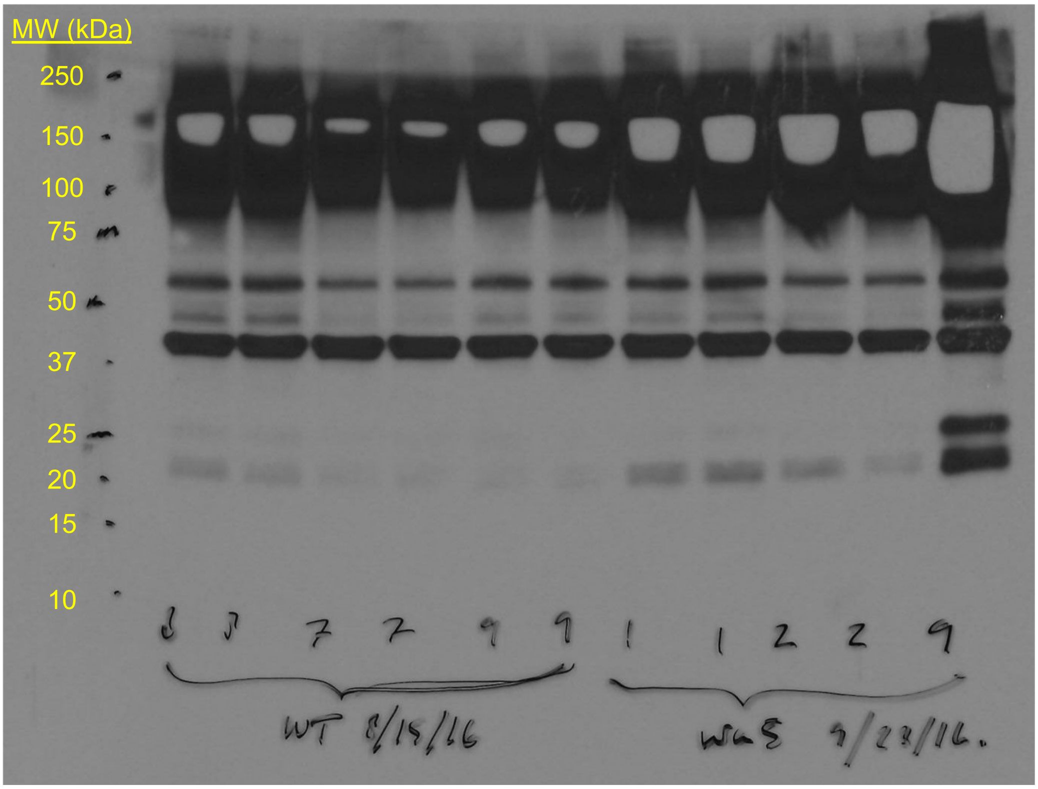

β-Actin (42 kDa)

WBs for Figure 3B

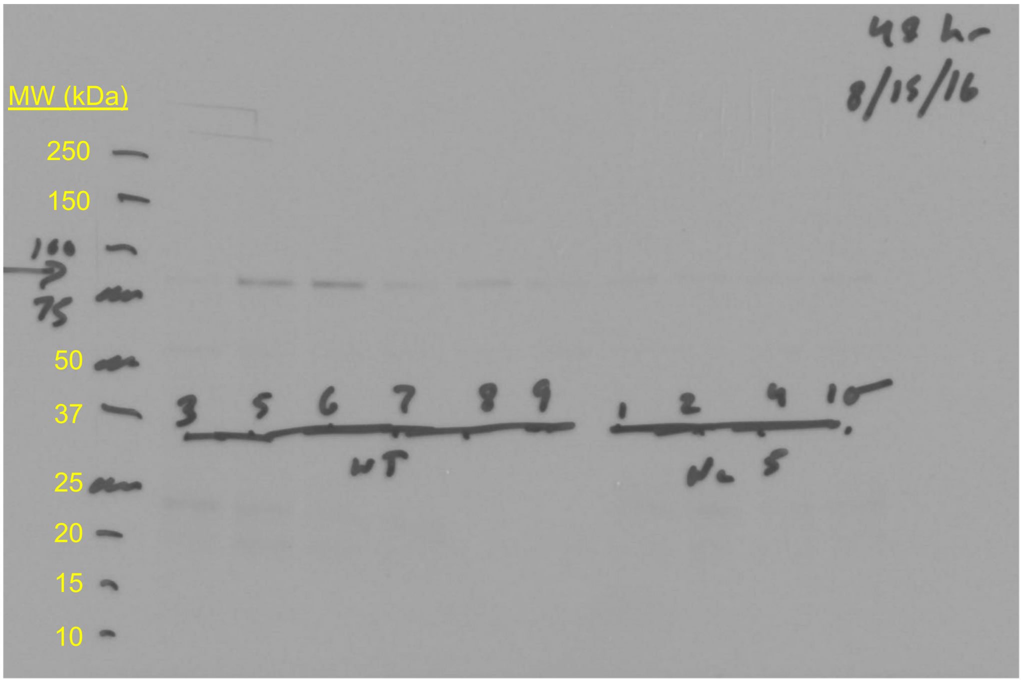

Cleaved PARP (89 kDa)

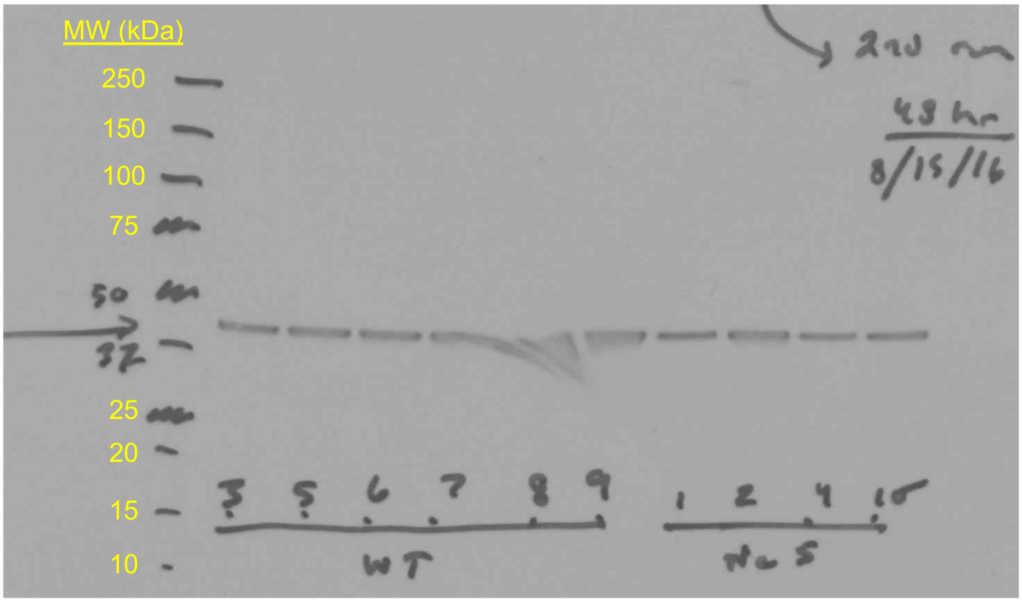

β-Actin (42 kDa)

WBs for Figure 4A

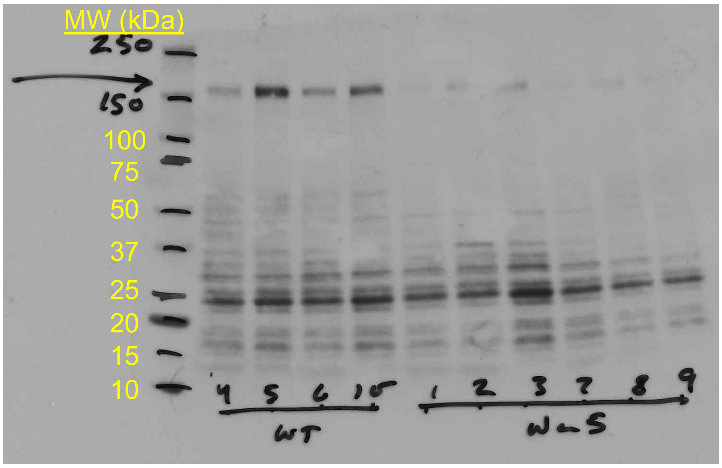

p-EGFR (175 kDa)

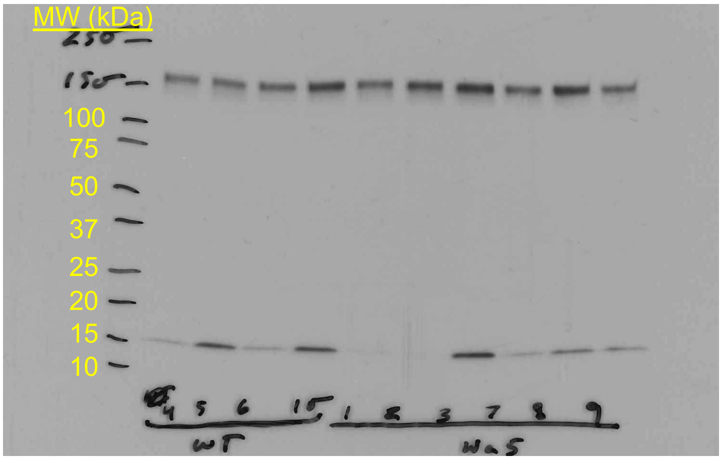

Total EGFR (175 kDa)

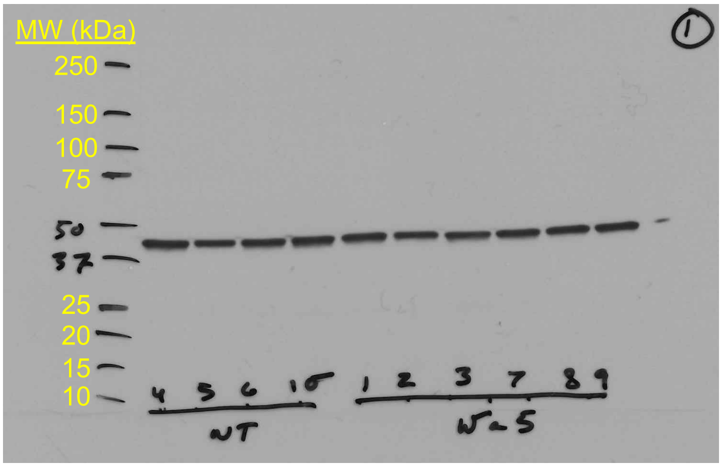

β-Actin (42 kDa)

WBs for Figure 4B

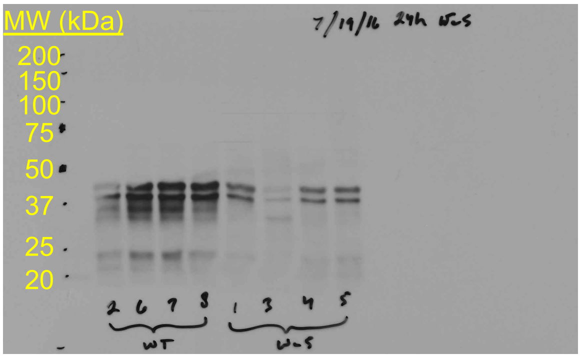

p-ERK1/2 (42, 44 kDa)

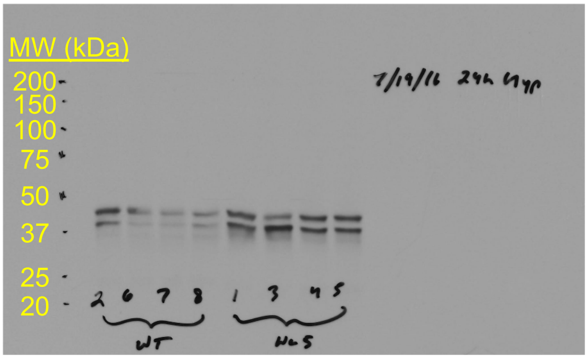

Total ERK1/2 (42, 44 kDa)

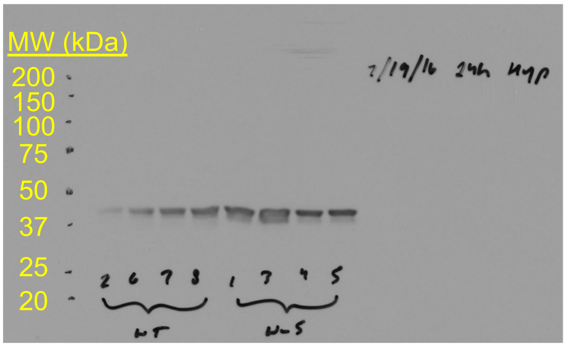

β-Actin (42 kDa)

WBs for Figure 4B (Not shown but included in densitometry)

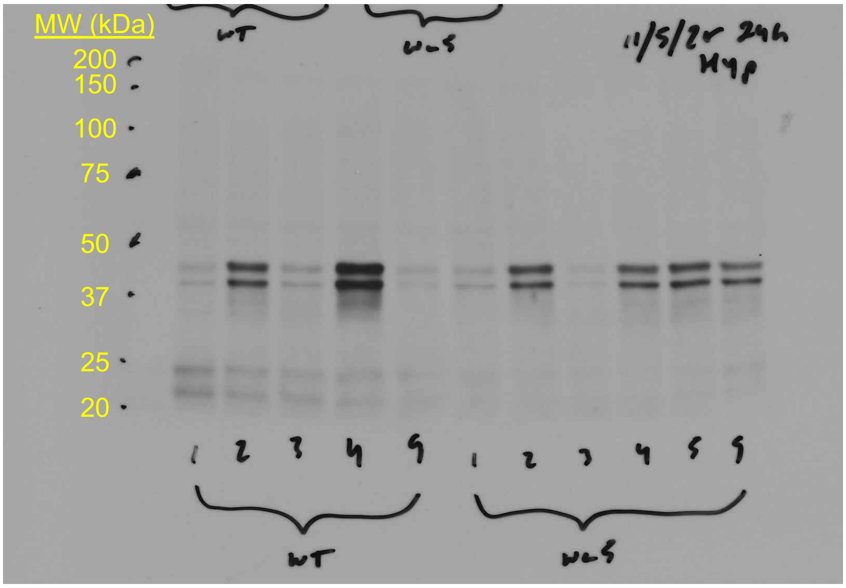

p-ERK1/2 (42, 44 kDa)

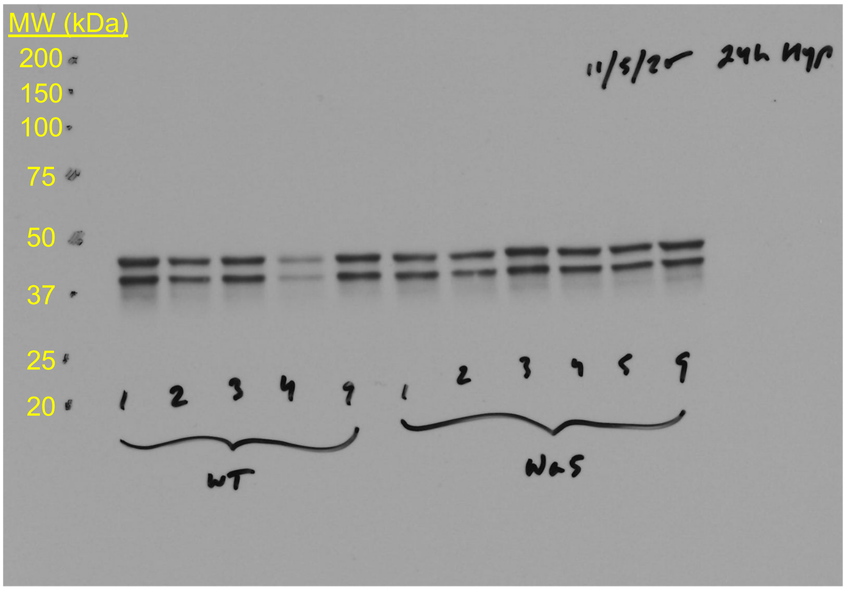

Total ERK1/2 (42, 44 kDa)

WBs for Figure 4C

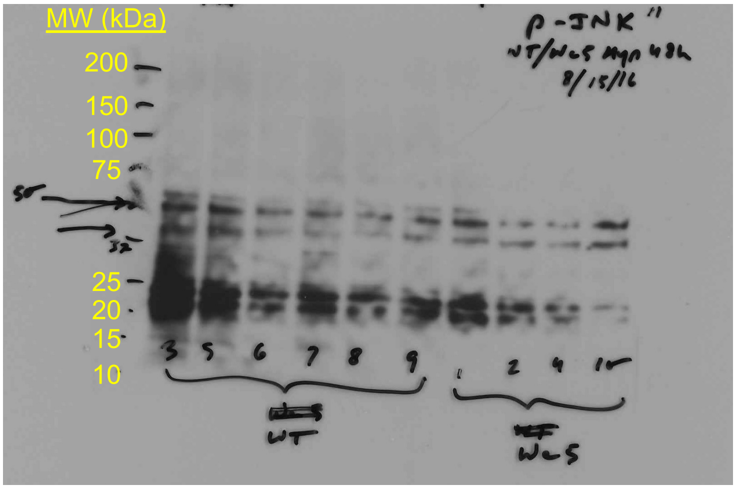

p-JNK1/2 (46, 54 kDa)

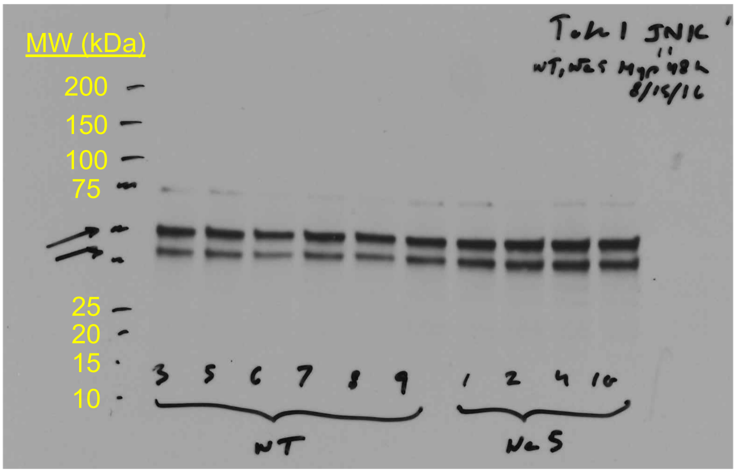

Total JNK1/2 (46, 54 kDa)

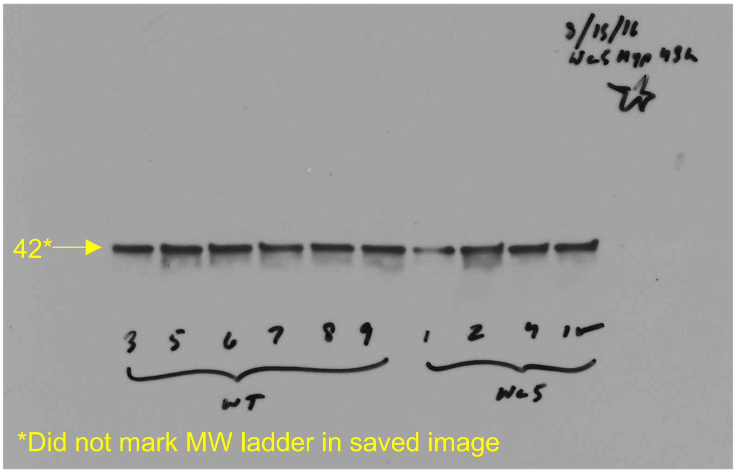

β-Actin (42 kDa)

WBs for Figure 4C

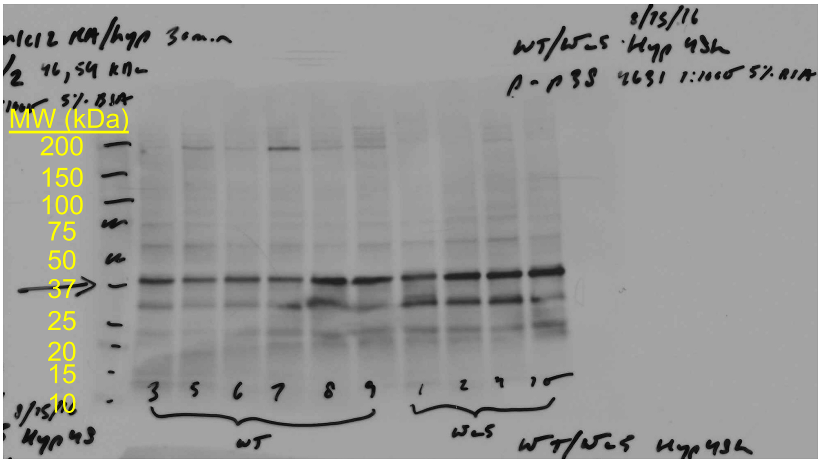

p-p38 (40 kDa)

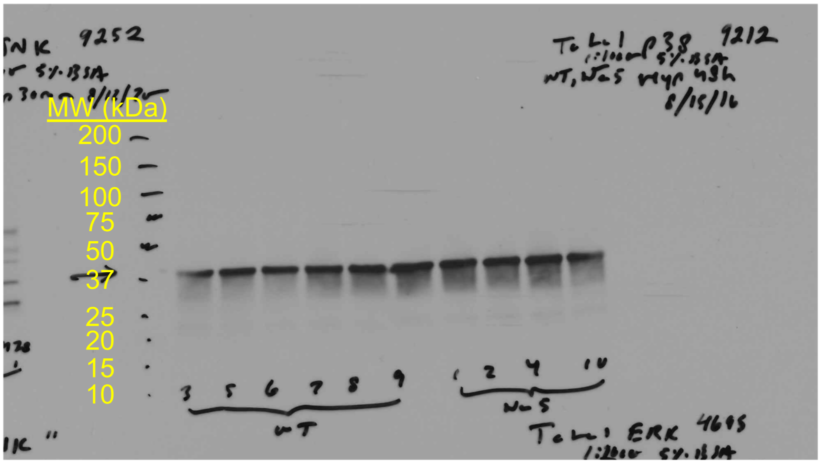

Total p38 (40 kDa)

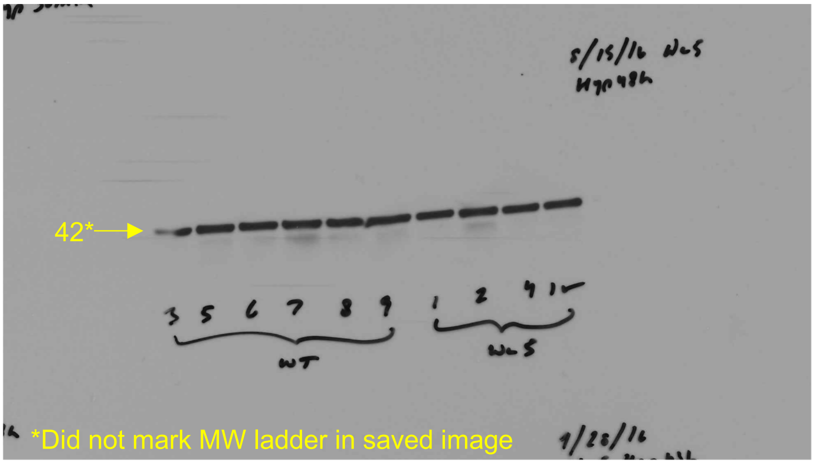

β-Actin (42 kDa)

WBs for Figure 4D

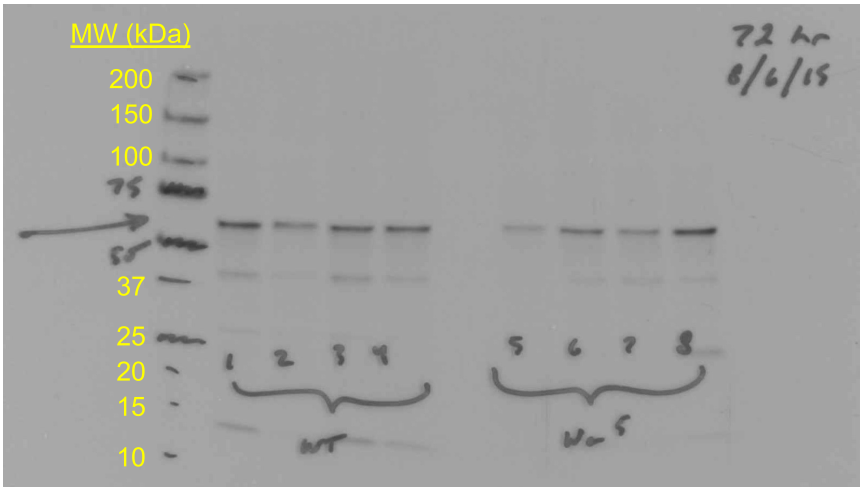

p-AKT (60 kDa)

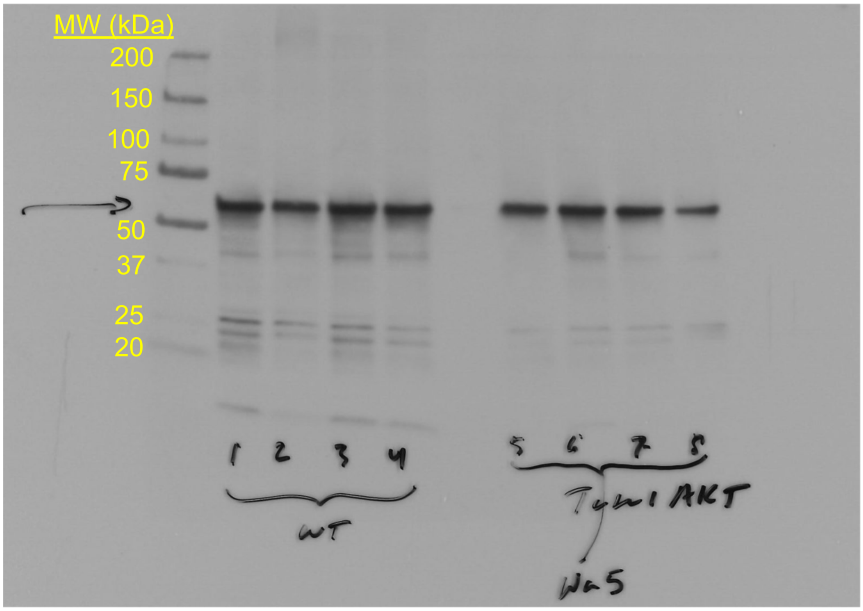

Total AKT (60 kDa)

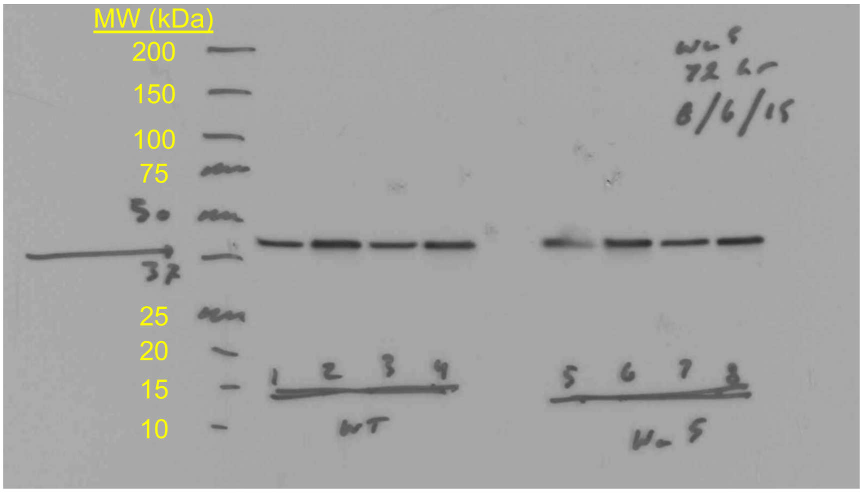

β-Actin (42 kDa)

WBs for Figure 5A

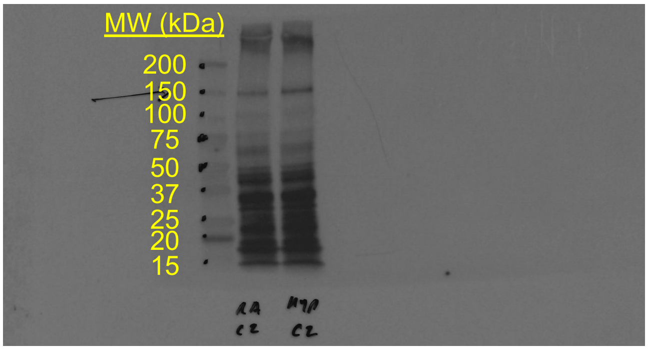

p-EGFR (175 kDa)

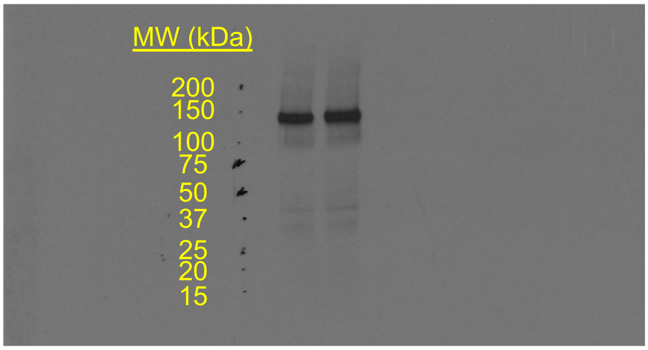

Total EGFR (175 kDa)

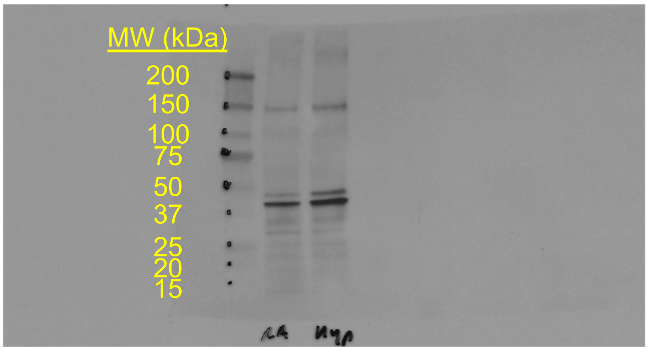

p-ERK1/2 (42, 44 kDa)

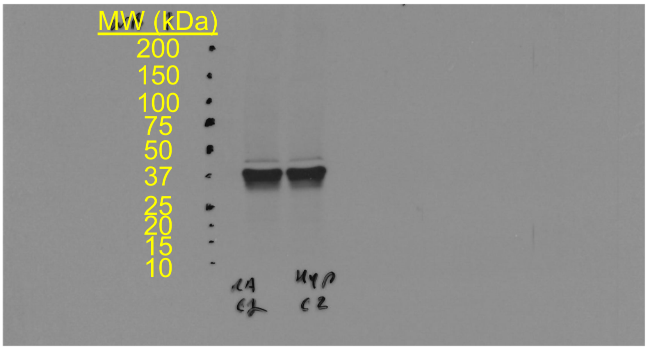

Total ERK1/2 (42, 44 kDa)

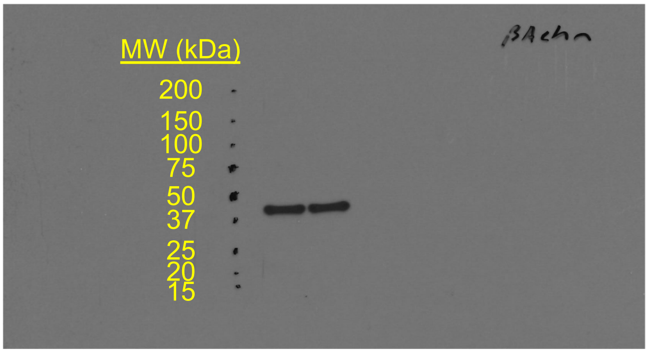

β-Actin (42 kDa)

WBs for Figure 5B

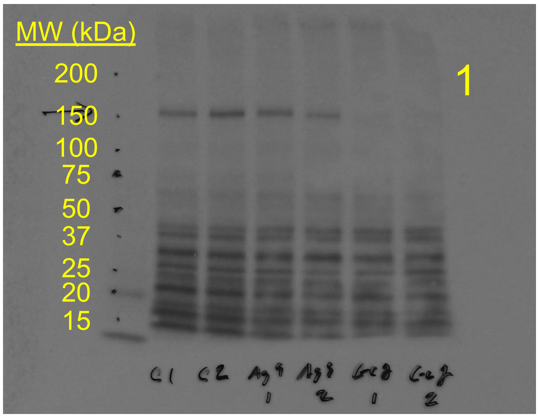

p-EGFR (175 kDa)

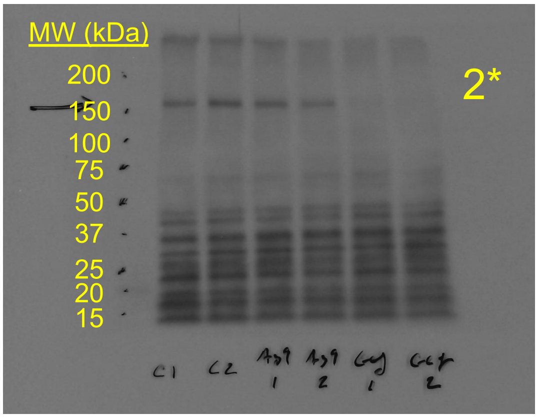

p-EGFR (175 kDa)

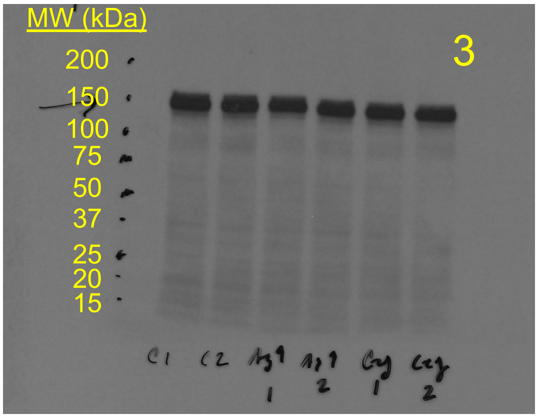

Total EGFR (175 kDa)

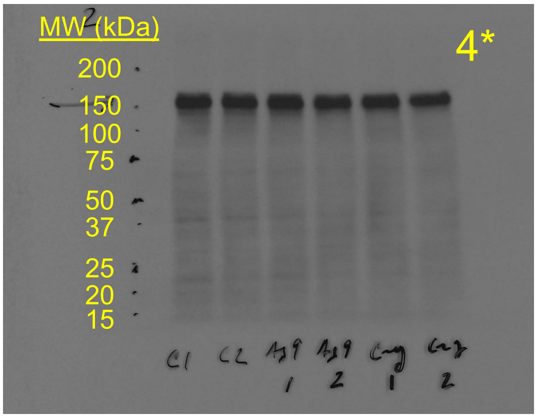

Total EGFR (175 kDa)

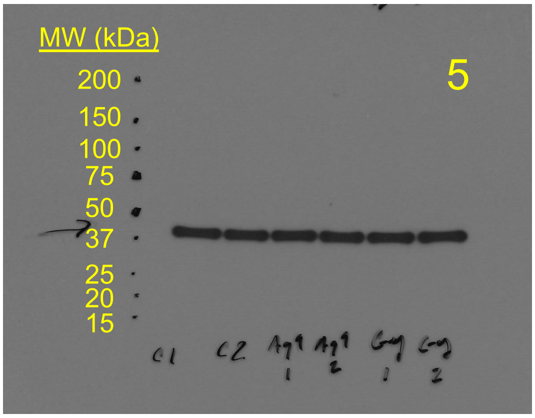

β-Actin (42 kDa)

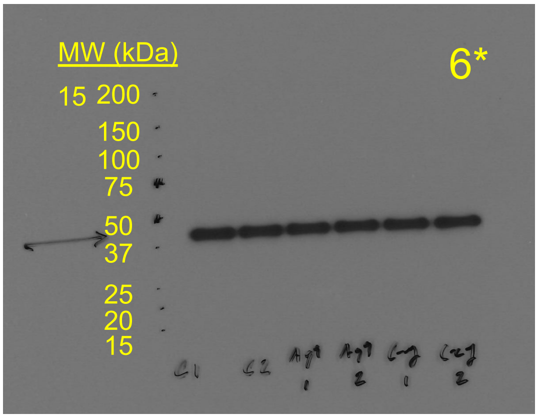

β-Actin (42 kDa)

*WBs 2, 4, and 6 not shown but included in densitometry

WBs for Figure 5C

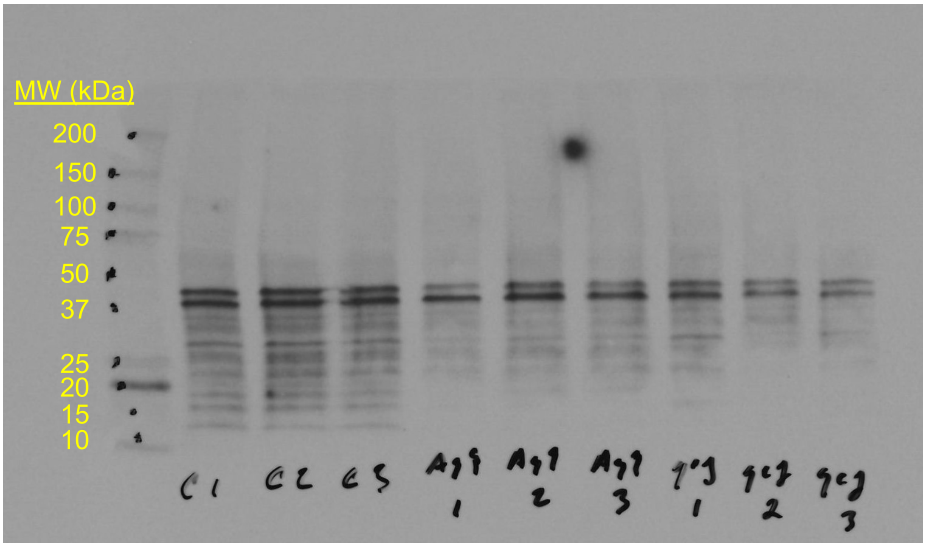

p-ERK1/2 (42, 44 kDa)

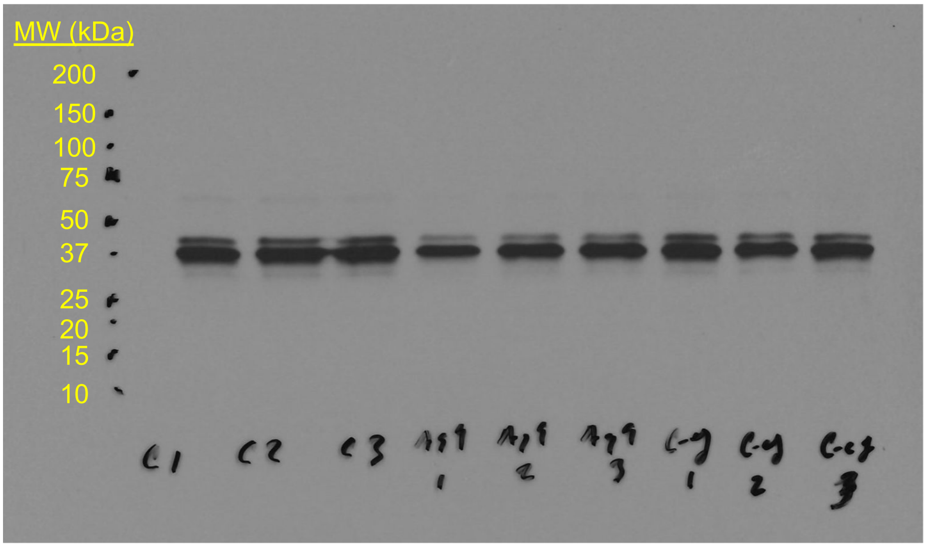

Total ERK1/2 (42, 44 kDa)

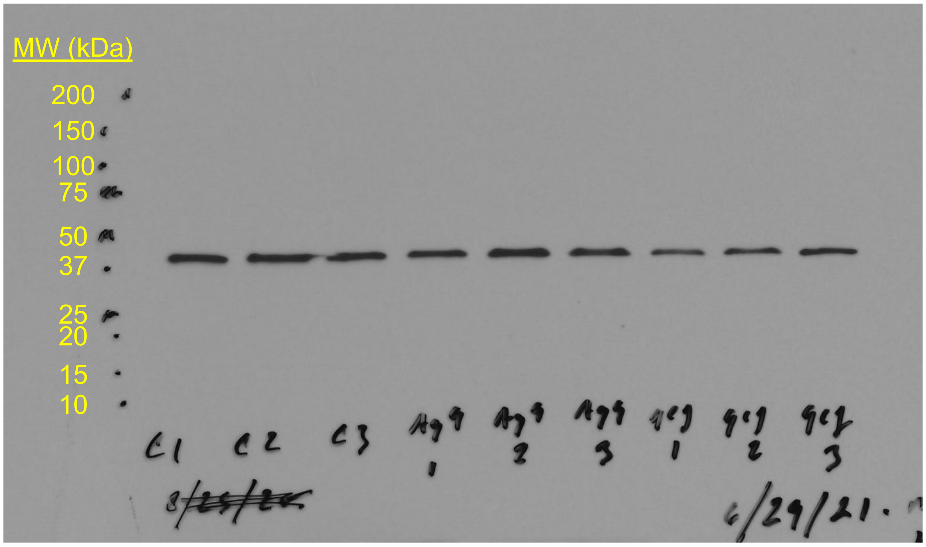

β-Actin (42 kDa)

WBs for Figure 6B

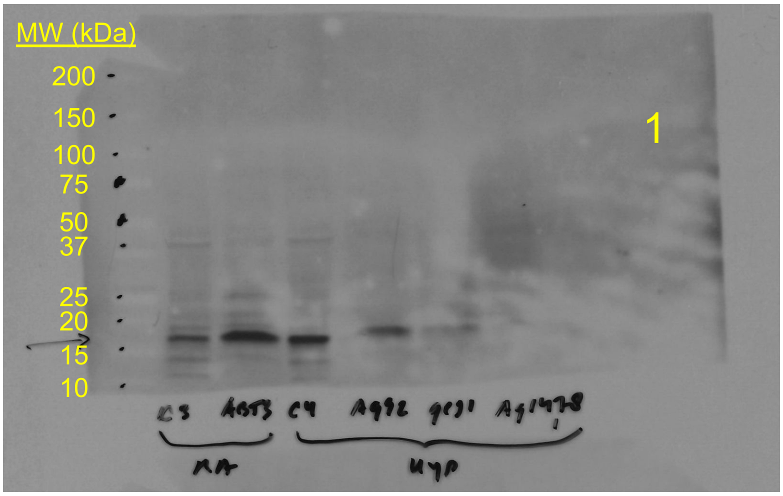

Cleaved caspase-3 (17, 19 kDa)

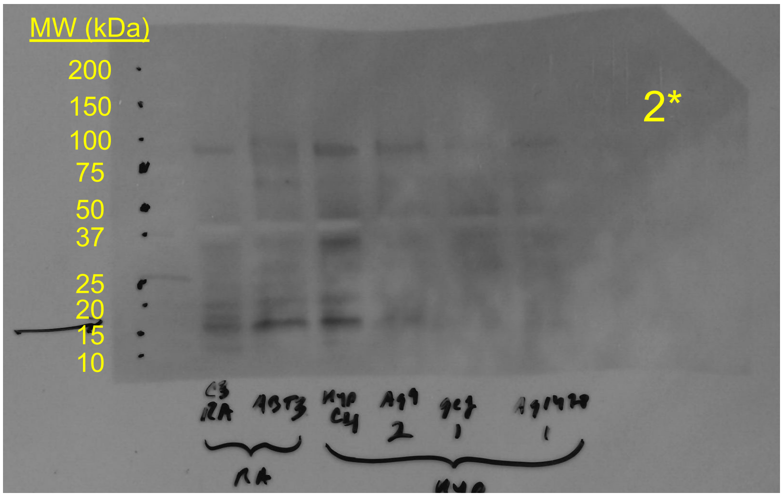

Cleaved caspase-3 (17, 19 kDa)

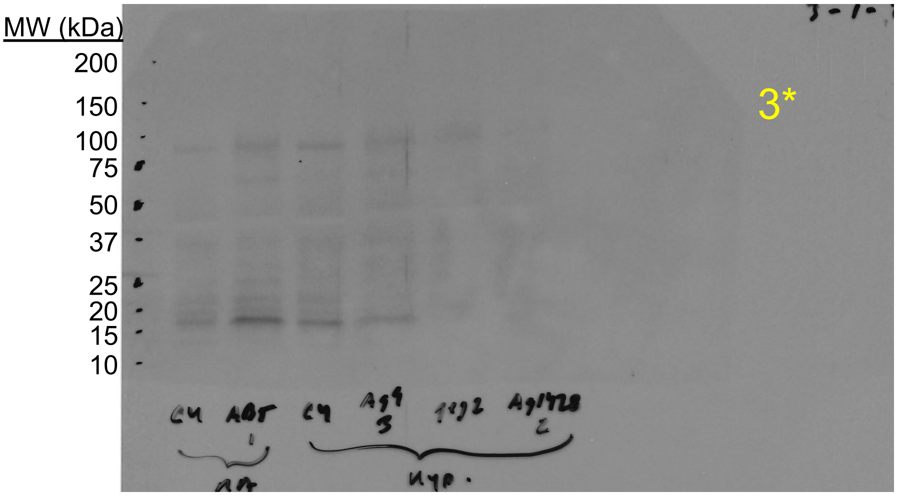

Cleaved caspase-3 (17, 19 kDa)

*WBs 2 and 3 not shown but included in densitometry

WBs for Figure 6C

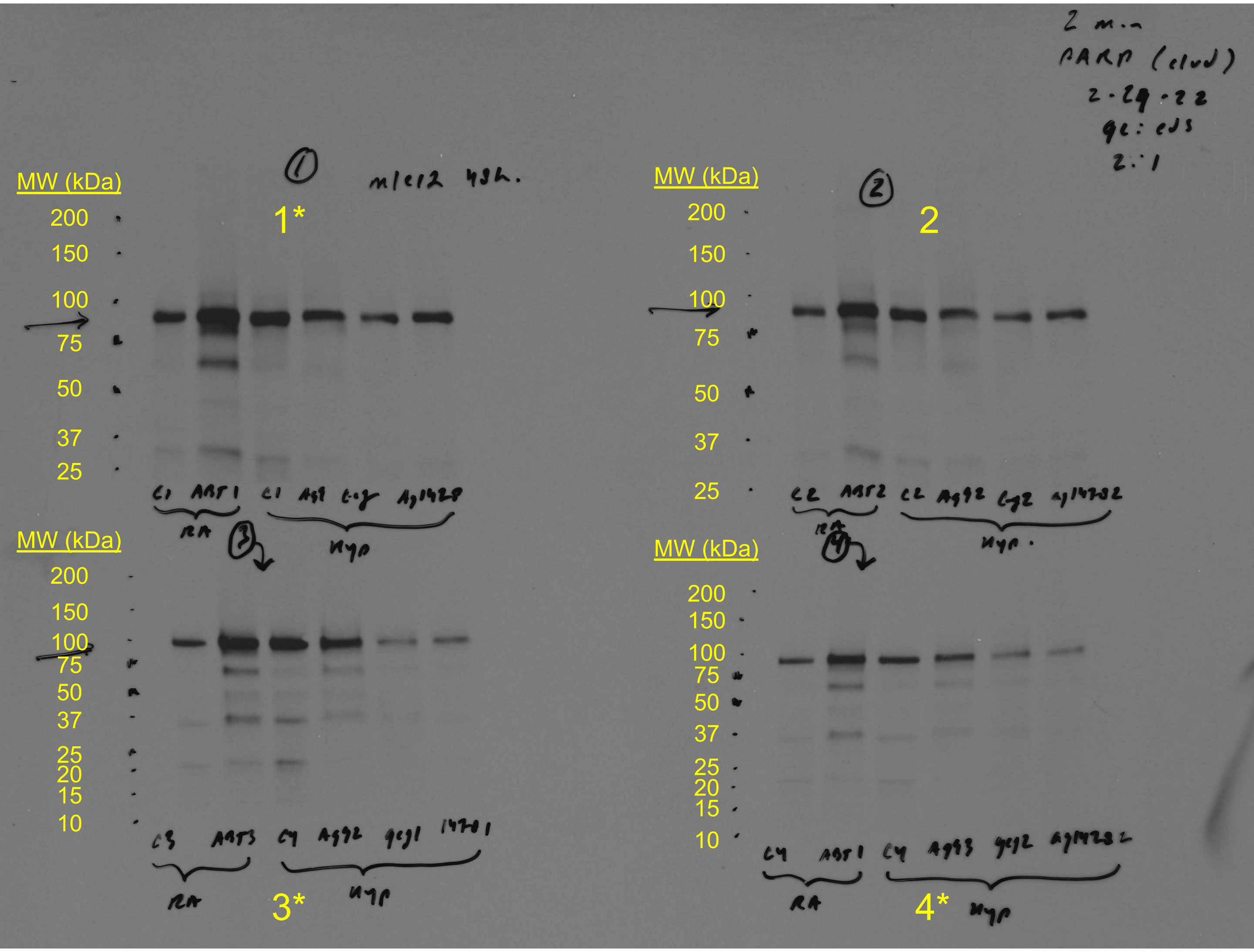

Cleaved PARP (89 kDa)

*WBs 1, 3, and 4 not shown but included in densitometry

WBs for Figure 6B

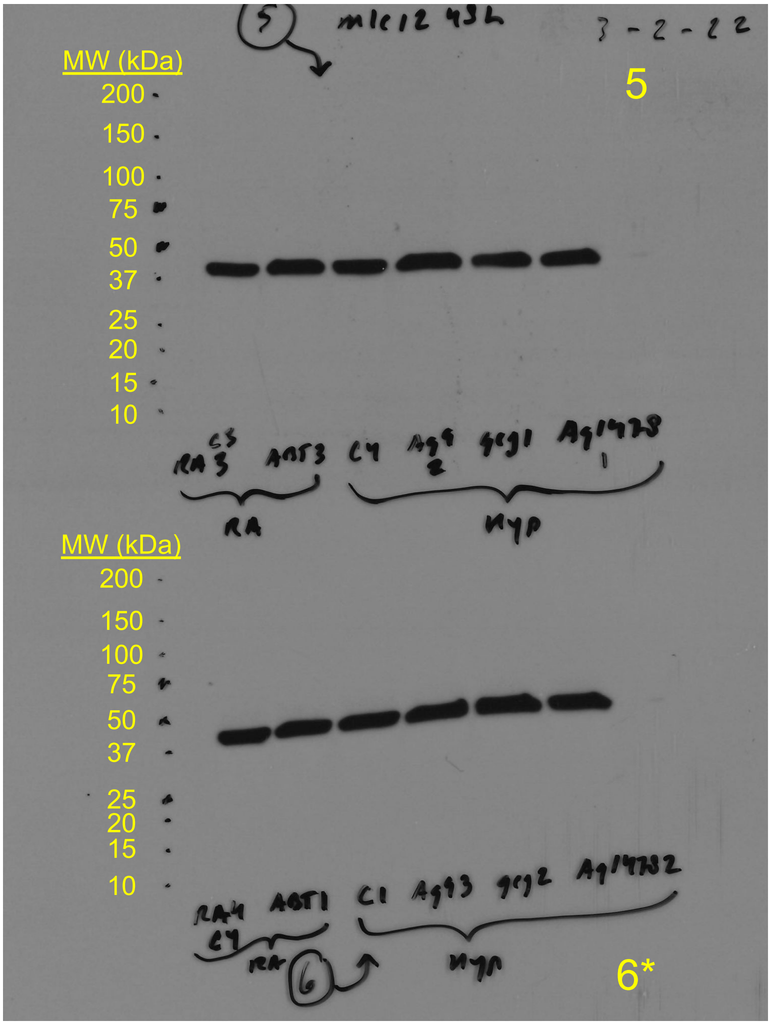

β-Actin (42 kDa)

*WB 6 not shown but included in densitometry

WBs for Figure 6B, 6C

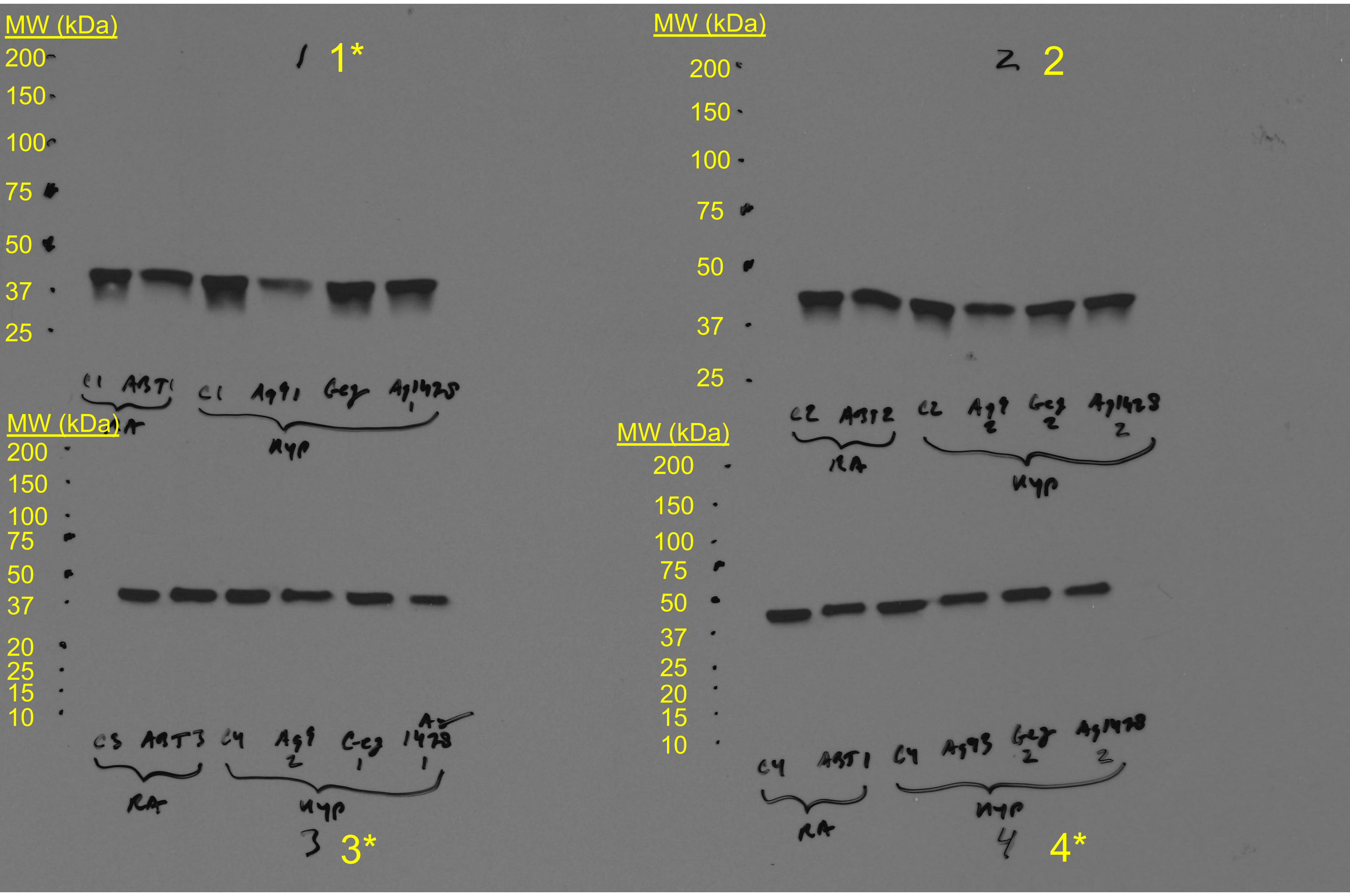

β-Actin (42 kDa)

*WBs 1, 3, and 4 not shown but included in densitometry

WBs for Figure 7A

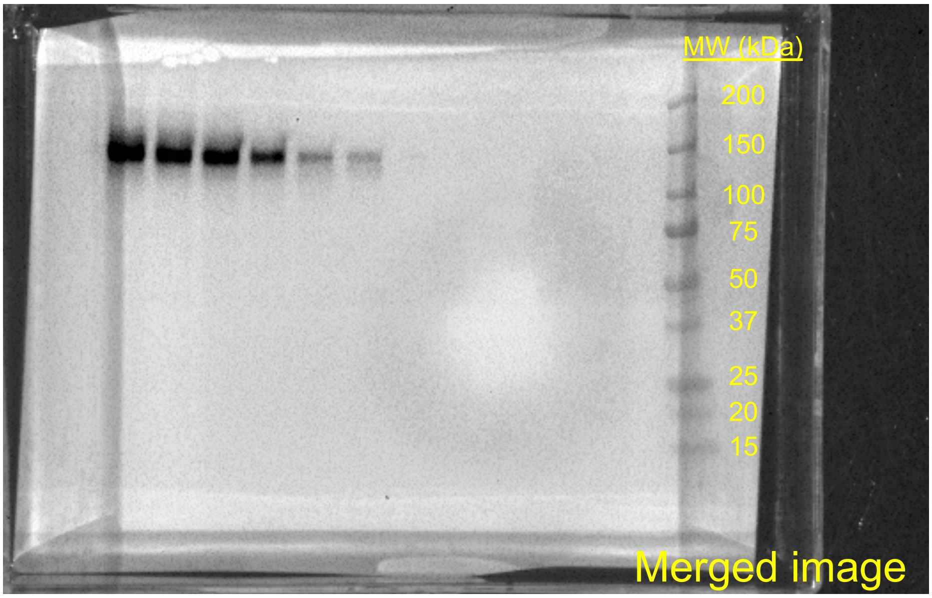

p-EGFR (175 kDa)

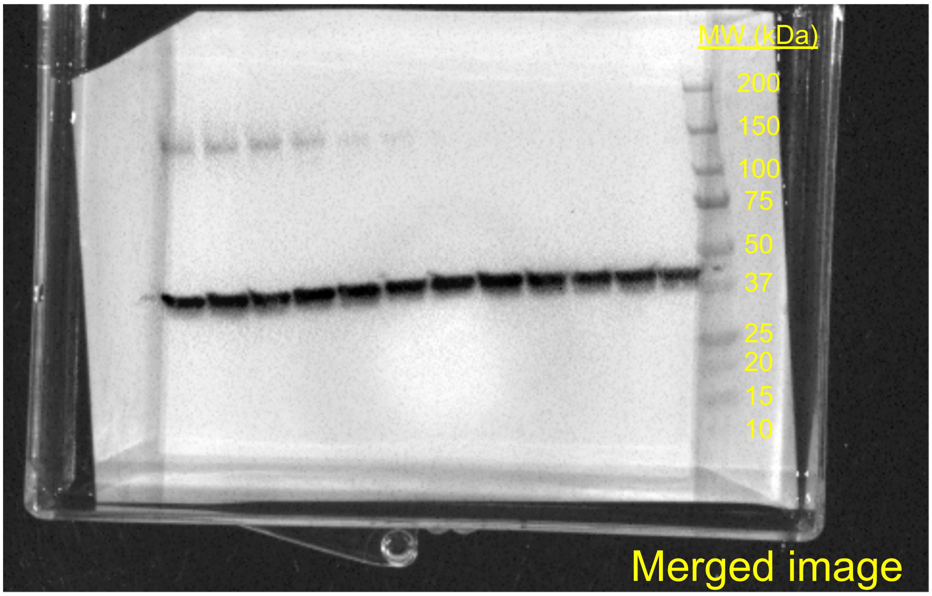

β-Actin (42 kDa)

WBs for Figure 7B

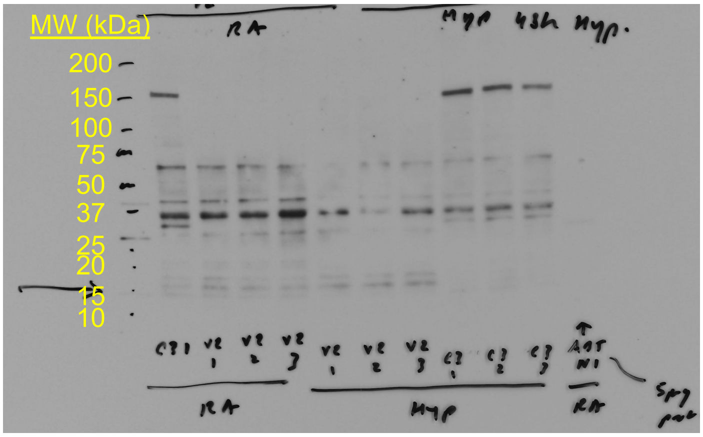

Cleaved caspase-3 (17, 19 kDa)

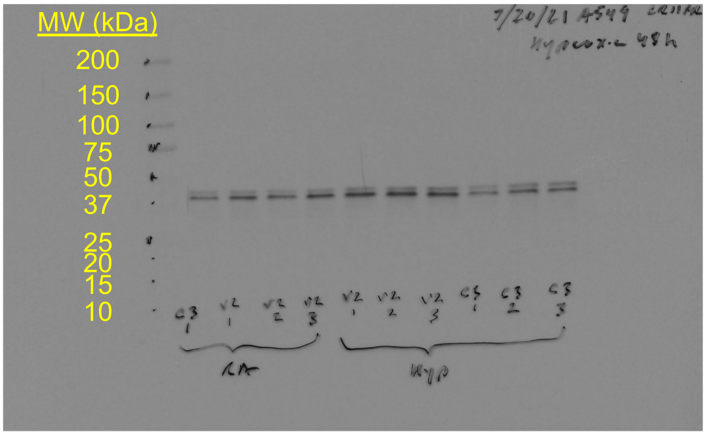

p-ERK1/2 (42, 44 kDa)

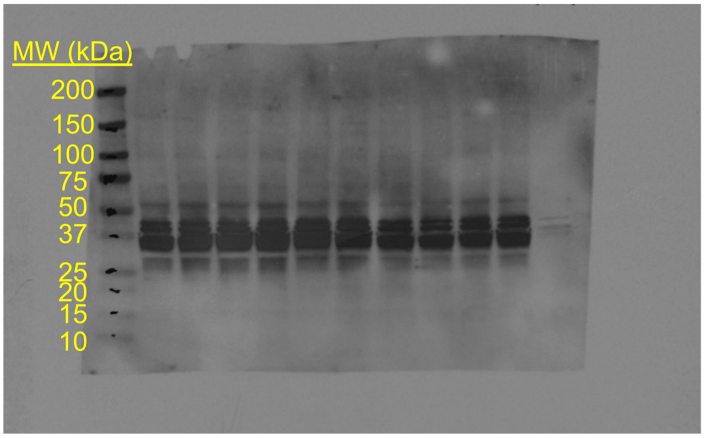

Total ERK1/2 (42, 44 kDa)

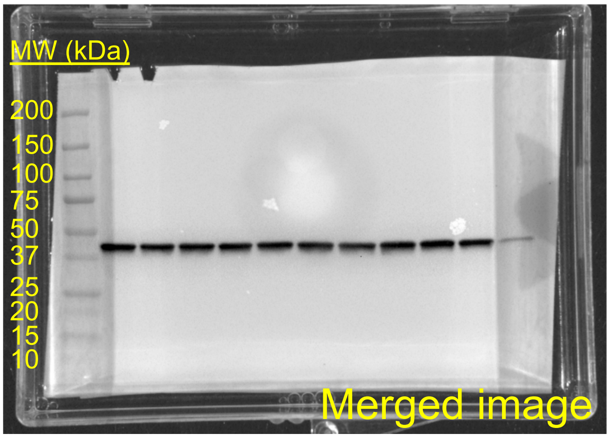

β-Actin (42 kDa)

WBs for Figure 7C

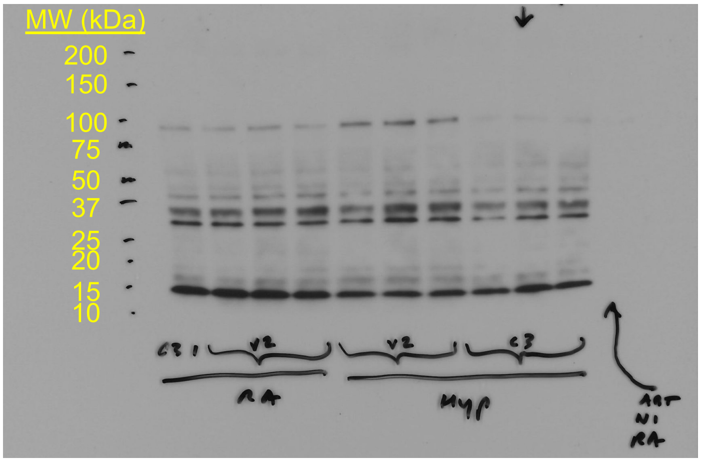

Cleaved PARP (89 kDa)

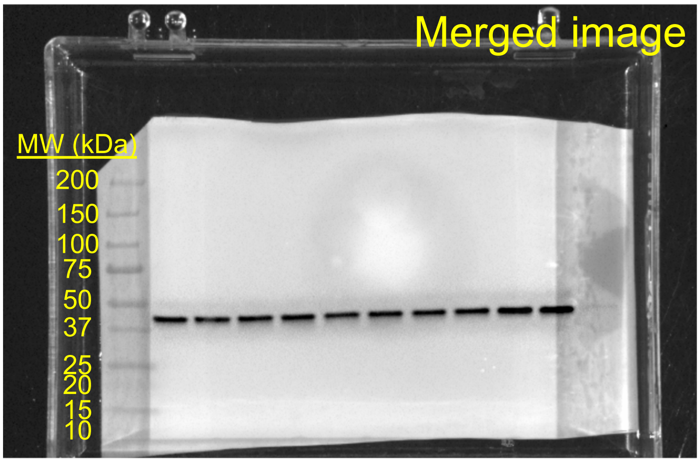

β-Actin (42 kDa)

WBs for Figure S.4

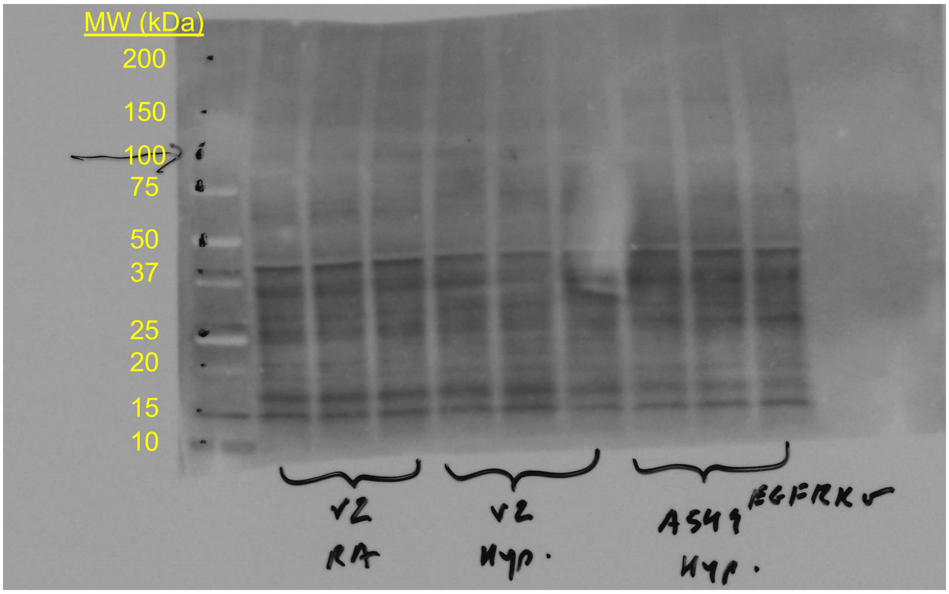

HIF-1α (120 kDa)

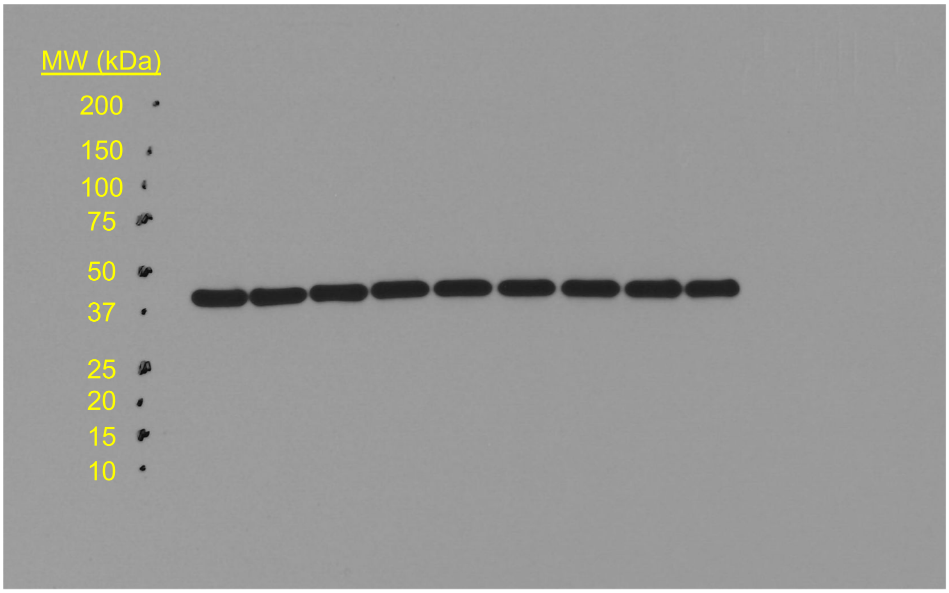

β-Actin (42 kDa)
